# Measuring premature and cumulative family member bereavement: Racial disparities and later mortality risk

**DOI:** 10.1073/pnas.2313600122

**Published:** 2025-06-10

**Authors:** Michelle Chang, Theodore F. Robles

**Affiliations:** ^a^Department of Psychology, University of California Los Angeles, Los Angeles, CA 90095

**Keywords:** loss, bereavement, race, disparities, mortality

## Abstract

Though racial disparities in shortened life expectancy have been well established, racial disparities in the burden of bereavement after such premature deaths are severely understudied. This is, in part, due to a lack of measurement tools for characterizing lifetime exposure to loss. We propose three indices that simultaneously quantify premature and cumulative lifetime loss—two typically unmeasured dimensions of loss. Using a longitudinal US sample of 27,985 participants from the Health and Retirement Study (1992 to 2020) who experienced at least one lifetime loss, hierarchical linear models accounting for participants nested within households showed that Black participants and Native American participants had higher premature and cumulative burden of family member loss over the lifetime than all other racial groups across all three indices. These effects remained for Black participants after controlling for covariates such as parental education, household size, and years in the study. Second, we found that loss burden at study enrollment prospectively related to all-cause mortality. Depending on prematurity, each additional loss related to higher odds of dying during the study period after controlling for covariates such as chronic health conditions. Together, our work leverages prospective, longitudinal methodologies to identify racial disparities in exposure to earlier and repeated death and its impact on mortality among the bereaved. The proposed measurement approach has future applications for understanding how loss exposure—both “too soon” and “too much”—predicts poor health and earlier mortality.

## Significance

Structural racism has created longstanding conditions for certain racially oppressed groups to die prematurely. While premature death is studied as an end point, considerably less work studies it as a “beginning point” for surviving bereaved communities. Exposure to earlier and repeated deaths is an important lens for characterizing suffering in people of color, and we create three measures of such loss exposure. Results showed that Black and Native American participants lost family members “too soon” and “too much” compared to all other participants. Moreover, those with higher loss burden had higher odds of dying during the study. Given crises of COVID and police brutality, this study urges attending to racial disparities in bereavement and mortality as products of structural racism.

Structural racism has created intergenerational conditions that contribute to longstanding inequities in chronic disease deaths and shortened life expectancy among people of color (1). Inaccessible healthcare, residential segregation, food insecurity, and incarceration contribute to cardiovascular disease and cancer burden as leading causes of death among Black Americans and Native Americans (2–4). While mortality is often thought of as an end point, it is also a “beginning point” for the surviving families and communities who must live with the ripple effects of bereavement. Although racial minorities experience greater risk of familial death earlier in life and higher rates of multiple deaths compared to their White counterparts (5, 6), exposure to death in communities of color remains severely understudied. Bereavement predicts multiple deleterious outcomes including greater substance use, physical illness, depression, and risk for earlier death (7). Prior work focuses on predominantly White and higher-SES samples and often fails to systematically report participant race (8–10). When experiences of loss in people of color are studied, deficits-based framing identifies their losses as violent and traumatic and their grief as “deviations” from default White participants’ (11). Taken together, racially oppressed communities are subject to a higher burden of loss over the lifetime, but characterizing inequities in this toll is long overdue.

Researchers have called for a life course perspective that links the timing and prematurity of event exposure to health inequities due to age-patterned exposures (12). Complementing this, cumulative stress models posit the sheer quantity of stressors has an additive effect (13). While prematurity (5, 14, 15) and quantity (16) of losses are typically examined separately, quantifying and integrating both dimensions can delineate their combined burden. We propose and compare three measurement approaches that simultaneously quantify these two typically unmeasured dimensions of bereavement in social epidemiology research—experiencing losses “too soon” (prematurely) and “too much” (cumulatively) over the life course.

Losing loved ones too soon refers to either the deceased individual dying prematurely (e.g., dying at a young age or by violent/unnatural means) or the bereaved individual experiencing loss prematurely (e.g., grieving at a young age). Premature losses are distinct from other losses because they are perceived as preventable, more stigmatizing, and unresolved (17, 18). Exposure to death during early sensitive periods may program stress circuitry, emotion regulation processes, and other mechanisms related to psychopathology (19). Communities of color also face a higher burden of premature losses. Black Americans are more likely to lose a family member at every decade of life (5), Black children are more likely to lose a caregiver (20), and Black and US-born Hispanic parents are more likely to lose a child (21) than White counterparts. Compared to White children, Native American youth have higher odds of losing a parent by age 17 (22). Current work typically measures loss during a single stage of life such as late adulthood (23) and overlooks many deaths that inherently occur too soon, such as an adult’s loss of a close sibling well before their projected life expectancy. As a result, many potentially devastating and premature losses receive substantially less attention (10).

Losing loved ones too much refers to cumulative exposure to deaths. For example, a 21-y-old Black man and 3-time homicide attempt survivor stated, “I’m tired of going to funerals” (24). People grieving multiple losses simultaneously due to contexts of war, disaster, or accidents have a worsened mental illness prognosis compared to those grieving only one loss (25, 26). However, that work focuses on losses from a single, recent event and rarely measures histories of multiple prior losses. Moreover, studies often binarily record number of losses as “1” or “2+,” lacking differentiation between two versus three losses (16, 27). Cumulative stress models propose that exposure to adversity has additive effects leading to greater risk for disease and all-cause mortality (13). For example, middle-aged Black women who experienced at least three upsetting deaths had a greater risk of cardiovascular disease as measured by greater carotid intima-media thickness (28), and Black participants with multiple family member deaths had a greater increase in cardiometabolic conditions as they aged (29). While no quantitative research has yet examined burden of loss among Native Americans, qualitative results support the lived experiences that Native Americans experience deaths of loved ones more frequently, contributing to mental health concerns with depression and PTSD (30).

Integrating premature and cumulative exposure allows for a more comprehensive characterization of the burden of life course loss. First, solely counting the presence or absence of adverse events has not provided insight into the underlying mechanisms linking event exposure to the incidence and variability in deleterious outcomes (31). Consistent with lifespan developmental theories and the biopsychosocial model, we can organize risk and protective factors, as well as health outcomes, by developmental contexts that unfold across the lifespan (32, 33). Additionally, measuring premature and cumulative exposure dimensions separately may overlook their inherent overlap and shared variance. These dimensions of loss, along with their antecedents and impacts, may also be distinct from other commonly measured lifetime stressors (e.g., parental substance use) and chronic stressors (e.g., ongoing health problems).

Moreover, after the death of a loved one, those who are left grieving are themselves more susceptible to dying prematurely—thus contributing to cycles of premature loss and premature death. Loss of a close family member such as a child or spouse is robustly associated with between 1.5 and 3.6 times greater risk for earlier mortality across population-based studies in the United States (15), Iceland (34), and Finland (35). Premature loss also prospectively relates to earlier mortality (15, 35, 36), though existing research studies predominantly White samples in the United States and Europe in the context of one type of loss (i.e., widowhood) (36, 37).

Using a population-based US sample, this study first elucidates racial disparities in exposure to earlier and repeated loss of close family members over the lifespan. We propose three indices to measure premature and cumulative lifetime loss. We evaluated their construct validity as measures distinct from existing measures of lifetime stress and chronic stress. We hypothesized that racial minority participants experienced more family deaths throughout their lifetimes and experienced these deaths at earlier ages than White participants. Due to the heterogeneity in racial disparities by cause of death in the United States, such as higher rates of death in Latine groups for diabetes and kidney diseases compared to White groups (38) and worse outcomes among Pacific Islanders compared to Asians (39), we chose to broadly hypothesize about the increased burden of loss among racial minorities. However, we predicted that this relationship would be most pronounced in Black and Native American participants, both of whom consistently have lowest life expectancies among US racial groups. Second, we examined whether cumulative and premature loss at study enrollment (as measured by our indices) predicts all-cause mortality. We hypothesized that a higher lifetime burden of loss would predict earlier death during the study period among the bereaved. We also stratified analyses by race to explore whether these relationships were stronger in people of color.

## Materials and Methods

### Data.

This project utilized data from the longitudinal Health and Retirement Study (HRS) maintained by the National Institute on Aging. This nationally representative survey of US adults over the age of 50 assessed participants every 2 y to follow changes in their health, employment, and social structures across 28 y (1992-2020). HRS oversamples for underrepresented Black and Hispanic participants (40) and is the only US population-based dataset that assesses lifetime exposure to kin deaths, ages of exposure to deaths, and ages at which the deceased died. Data are publicly available (*SI Appendix*, Table S11) or available to access by application (https://hrs.isr.umich.edu/data-products).

### Participants.

We selected a sample of 42,389 participants over the age of 50 who had at least 1 kin loss recorded in HRS. We excluded 14,404 participants who had no kin losses reported due to our inability to confirm whether these participants represented 0 kin losses or missing data, in following our preregistered analytic plan for sample selection (https://osf.io/3j84a, https://osf.io/6mxja). Those with no recorded losses had characteristics suggesting that the recording of no losses could possibly represent data that were missing not at random. In the excluded sample with no losses, 43% were from the oldest possible birth cohort whereas only 6% of those with at least one loss were from the oldest birth cohort (*SI Appendix*, Table S1). Moreover, a significant portion of the excluded sample was lost to attrition; 61% of the excluded sample died by 2020 compared to 31% of the included sample (*SI Appendix*, Table S1). Compared to the excluded sample with no kin losses, our final analytic sample (*n* = 27,985) who had experienced at least 1 kin loss was more likely to be from a later birth cohort, have a younger age, survive until 2020, and report higher parental education, higher household capital income, and a larger household size at baseline (*SI Appendix*, Table S1).

### Variables.

For the loss indices, respondents’ ages of exposure to the deaths of a biological mother, father, brother, sister, daughter, son, and/or spouse were collected, in addition to either 1) month and year of the death, or 2) wave of the death. Based on participants’ birth month and year, we calculated participant age at exposure to kin death. For deaths with only wave of death recorded, the wave midpoint was approximated as the death date (e.g., a death recorded during the 2008 wave was approximated as having a June 2007 death date). The deceased’s age at death was available for spouses, parents, and children, but not for siblings. Sibling and child deaths were collected at the household level and thus may be underreported in HRS. Sibling deaths were also not assessed after both parents were reported deceased, and date of sibling death was not assessed at many waves.

A lifetime trauma checklist (41) summed experiences of death of a child, natural disaster, combat, substance addiction, assault, and life-threatening conditions, and a childhood trauma checklist (41) summed experiences of repeating a year of school, trouble with police, parental substance use, and parental physical abuse before age 18. For more recent life events, we summed the occurrence of job loss, unemployment (participant or household), move to a worse neighborhood, and robbery in the past 5 y (42). A list of 8 chronic stressors from the past 12 mo included ongoing health problems, physical or emotional problems in a spouse or child, familial substance use, difficulties at work, financial strain, problems in a close relationship, or caregiving; only those rated “Yes, very upsetting” (4) were summed (43).

Respondent race was recorded in a cross-wave file as White/Caucasian, Black/African American, American Indian, Asian American, Native Hawaiian, Pacific Islander, or Other. Participants also responded whether or not they were Hispanic. Following APA Style guidelines for reporting race and ethnicity, throughout this article we utilize “White” to reference whom HRS labels as non-Hispanic “White/Caucasian,” “Black” to comprehensively reference people of the African diaspora, “Hispanic” to reference Hispanic and White Hispanic individuals, and “Native American” to reference whom HRS labels as “American Indian” and “Native Hawaiian.”

Participant mortality was based on whether participants were recorded as alive or deceased at the most recent wave of data collection in 2020, as well as their age at death if applicable. *SI Appendix*, Table S11 lists all survey questions and their corresponding datasets and variable names.

### Covariates.

Covariates were based on prior work using HRS data (15, 21), including participant sex and several variables recorded at baseline (study enrollment): participant age, household size, number of times married, number of living siblings, number of children ever born, and whether the Census region of participant residence was in the South. SES-related covariates included respondents’ family financial situation from birth to age 16 (1 = pretty well off financially; 4 = poor), mother’s and father’s years of education completed, and household capital income at baseline.

For models of racial disparities in lifetime loss, we also controlled for participant death and time in the study. To account for the possibility that participants’ lifetime loss scores may be lower due to their own premature deaths, we controlled for whether or not participants were deceased by the 2020 wave. To account for the possibility that participants’ loss scores may be higher if they had been enrolled in the study for longer and thus had more years to experience losses, we controlled for the number of years enrolled in the study.^*^

Health-related covariates used only for models of loss burden and all-cause mortality included smoker status, drinking, number of lifetime chronic conditions (up to 8 chronic conditions including hypertension, diabetes, cancer, lung disease, heart problems, stroke, mental health problems, and arthritis), and general health rating (1 = excellent, 5 = poor).

### Analyses.

#### Loss indices.

We computed three indices to capture the prematurity and quantity of deaths grieved over the lifespan (*SI Appendix*, *Methods*). The “bereaved-dependent” and “deceased-dependent” distinction in our indices refers to whether the data on loss timing is derived from the bereaved participant’s age when the loss occurred, or from the deceased family member’s age at death, respectively—both of which may have different impacts depending on life stage. For example, early life or childhood (ages 0 to 17) is marked by substantial change; losses occurring in this life stage are the most premature and have short and long-term consequences to stress response and reactivity (44). Losses occurring during emerging adulthood (ages 18 to 29) may exacerbate the identity struggles, instability, increased independence, and low social support typical to this period (45). Characterized by increasing well-being but decreasing functions, middle adulthood (ages 30 to 50) bereavement is both subject to the influence of early life histories of loss and predictive of later-life outcomes (46). Finally, bereavement or dying during older adulthood (ages 51+) is a frequent occurrence, though its toll depends on various relational and contextual factors (47).

While age-related cutoffs for life stages vary greatly across researchers and are relative approximations, the life events and roles that define these periods serve as benchmarks for categorizing age (48). We determined these age-based cutoffs based on theoretical background and empirical evidence from bodies of literature such as the Adverse Childhood Experiences (ACEs) literature, the Midlife Development in the US (MIDUS) survey, and the Health and Retirement Study (HRS). Detailed rationale for defining each life stage is in *SI Appendix*, *Methods*.

##### Bereaved-dependent childhood losses (BCL) index.

The BCL index only counts the number of earliest losses, consistent with ACEs research. Losses occurring between 0 and 17 y old were coded as 1, while all other losses occurring later were coded as 0.

##### Bereaved-dependent life stage (BLS) index.

The BLS index counts all lifetime losses. Losses are weighted by the prematurity of the bereaved’s developmental stage during loss exposure: 4 (childhood, ages 0 to 17), 3 (emerging adulthood, ages 18 to 29), 2 (middle adulthood, ages 30 to 50), and 1 (older adulthood, ages 51+).

##### Deceased-dependent life stage (DLS) index.

The DLS index alternatively conceptualizes prematurity of loss in terms of the deceased’s age at death. Losses are weighted by the prematurity of the deceased’s developmental stage at their death, assigned a higher score when the deceased was younger, and a lower score when older. The range of scores for each loss is the same as the BLS index, between 1 and 4.

#### Models.

For Aim 1, we assessed racial differences in premature and cumulative exposure to death over the lifetime. We conducted two-level multilevel linear models, with a random intercept to account for participants nested within households. For each of the three loss indices, we tested the unadjusted model without covariates and adjusted model accounting for covariates—yielding 6 total models. The predictor variable was participant race, and the outcome variable was lifetime loss index based on all recorded deaths in a participant’s life. To avoid specifying a reference group as the “default” race against which all other races would be compared, we computed binary contrasts that compared a particular racial group to the weighted mean of all other racial groups (49) and used the Bonferroni correction to adjust for multiple comparisons.

For Aim 2, we assessed the relationship between lifetime loss burden at baseline (i.e., participant’s enrollment in the study) and all-cause mortality in the larger sample using Cox mixed effects models. For each of the three loss indices, we tested an unadjusted model without covariates and an adjusted model controlling for covariates. Second, we ran race-stratified models. In following HRS guidance for analyses involving longitudinal data, we did not apply sampling weights because base-year weights do not account for participants entering the sample at different waves, and terminal-year weights do not correct for serious biases due to attrition (50). The adjusted models for Aims 1 and 2 are delineated in *SI Appendix*, *Methods*.

## Results

### Participants.

The 27,985 participants with at least 1 recorded kin loss in HRS comprised White (67.88%), Black/African American (17.81%), Hispanic (6.53%), Native American/Alaska Native/Native Hawaiian (2.60%), Asian American/Pacific Islander (1.40%), and Other (3.77%) participants. The mean baseline age in this sample was 57.42 y (SD = 7.46), and participant sex was reported as 57.25% female and 42.75% male.

### Lifetime Loss Index Descriptive Statistics and Correlations with Stressor Measures.

**Fig. 1.**
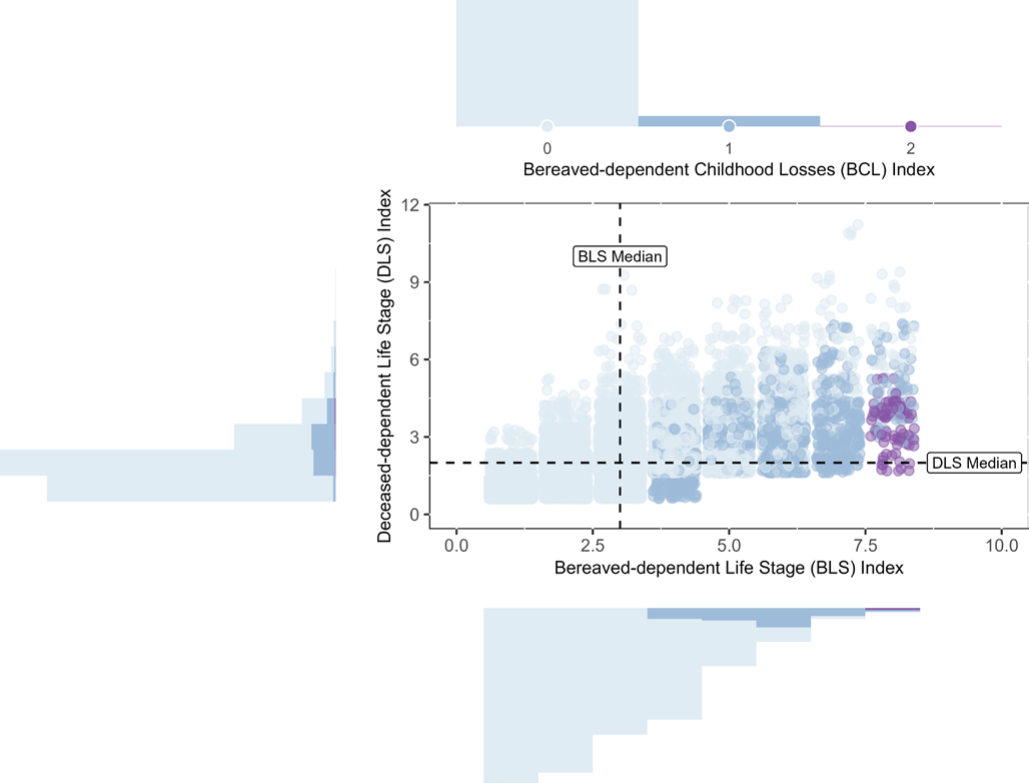
Marginal histograms depicting distributions of the three lifetime loss indices and a scatterplot depicting their interrelationships. Cells with less than 4 observations (i.e., BLS > 9) were excluded from the figure to comply with HRS data export guidelines. Points are jittered for ease of visualization.

**Fig. 2.**
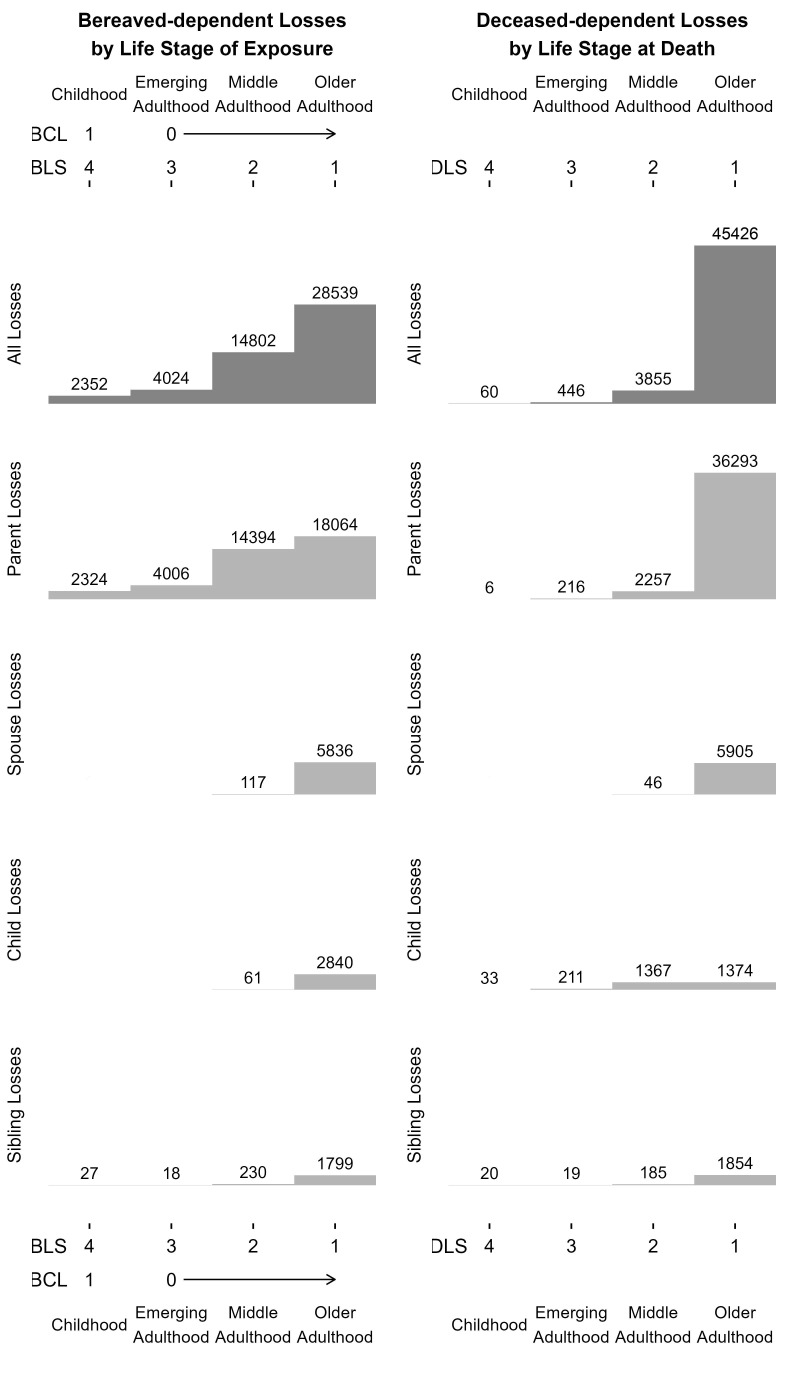
Distribution of scores assigned to each recorded loss according to three lifetime loss indices. Y-axis limits for the histograms for “All Losses” and “Parent Losses” are 46,000 and for the “Spouse Losses,” “Child Losses,” and “Sibling Losses” are 29,000. The gray shading visually distinguishes histograms for “All Losses” (dark gray) from histograms for each specific relationship’s losses (light gray).

Participants recorded a mean of 1.77 (SD = 0.77) kin losses grieved over the lifetime. Table 1 presents descriptive statistics for number of losses and all three lifetime loss indices, and *SI Appendix*, Table S2 presents descriptive statistics for the DLS index restricted to losses with exact age at death. Parent losses were most commonly recorded in this sample. Fig. 1 visualizes the overlap and differentiation in the three indices’ categorization of participant loss burden. Notably, the BCL index assigned a score of 0 to many losses that were counted in the BLS and DLS indices, meaning that the BCL index captured less information about loss burden. Fig. 2 depicts the life stages when losses occurred for the bereaved and deceased. In this sample, most losses across all relationships to the deceased occurred during participants’ late adulthood and when the deceased were in late adulthood.

**Table 1.** Psychometric properties of composite lifetime loss indices, stratified by relationship to deceased

		Quantity only	Prematurity only	Quantity and Prematurity	
		Number of losses	Bereaved-dependent childhood losses (BCL) index	Bereaved-dependent life stage (BLS) index	Deceased-dependent life stage (DLS) index
			(Each loss weighted by life stage, 1 = childhood, 0 = after childhood)	(Each loss weighted by life stage, 4 = earliest, 1 = latest)	(Each loss weighted by life stage, 4 = youngest, 1 = oldest)
All losses	*M*	1.77	0.08	2.84	1.96
	*SD*	0.81	0.29	1.65	1.07
	*Median*	2	0	3	2
	*N*	27,985	27,985	27,985	27,985
Parent losses only	*M*	1.39	0.08	2.43	1.48
	*SD*	0.71	0.29	1.69	0.82
	*Median*	2	0	2	2
	*N*	24,431	27,985	27,985	27,985
Spouse losses only	*M*	0.21	N/A	0.22	0.21
	*SD*	0.41		0.42	0.41
	*Median*	0		0	0
	*N*	5,952		27,985	27,985
Child losses only	*M*	0.11	N/A	0.11	0.17
	*SD*	0.39		0.38	0.67
	*Median*	0		0	0
	*N*	2,449		27,985	27,985
Sibling losses only	*M*	0.07	0.00	0.09	0.08
	*SD*	0.37	0.04	0.46	0.45
	*Median*	0	0	0	0
	*N*	1,426	27,985	27,985	27,985

All three lifetime loss indices had weak, positive correlations with lifetime trauma when excluding the item for child death (*r*s = 0.03-0.06, *P* < 0.001). The BCL and BLS indices, but not the DLS index, had weak, positive correlations with childhood trauma (*r*s = 0.03-0.05, *P* < 0.001) and recent stressors in the prior 5 y (*r*s = −0.03-0.05, *P* < 0.001). Moreover, there was little evidence of overlap in lifetime loss with chronic stressors over the past 12 mo (*r*s = 0.00-0.02). *SI Appendix*, Table S3 presents all Pearson’s correlations in detail.

### Aim 1: Does prematurity and quantity of family deaths experienced over the lifetime differ by race?

Table 2 presents all binary contrast results that compare each racial group to the weighted mean of all other racial groups, fitted to multilevel models. Across all unadjusted and adjusted models, Black participants reported higher lifetime burden of premature and cumulative loss compared to all other participants.

**Table 2.** Binary contrasts for multilevel models showing racial disparities in lifetime loss

	Number of losses		Bereaved-dependent childhood losses (BCL) index		Bereaved-dependent life stage (BLS) index		Deceased-dependent life stage (DLS) index	
Racial Group	Unadjusted	Adjusted	Unadjusted	Adjusted	Unadjusted	Adjusted	Unadjusted	Adjusted
Reference: Weighted grand mean of other groups								
White	−0.00 [−0.03, 0.03]	0.01 [−0.03, 0.05]	−0.03*** [−0.04, −0.02]	−0.01* [−0.02, −0.00]	−0.20*** [−0.27, −0.14]	0.02 [−0.06, 0.10]	−0.11** [−0.15, −0.07]	**−0.04^†^** [−0.10, 0.01]
Black	0.03 [−0.01, 0.07]	0.05* [0.00, 0.10]	0.03*** [0.02, 0.05]	0.02** [0.00, 0.03]	0.24*** [0.16, 0.31]	0.11** [0.02, 0.20]	0.15*** [0.10, 0.20]	0.12*** [0.06, 0.18]
Hispanic	0.00 [−0.05, 0.06]	−0.05 [−0.12, 0.02]	0.00 [−0.02, 0.02]	−0.00 [−0.02, 0.02]	−0.01 [−0.13, 0.10]	−0.17** [−0.31, −0.03]	**0.07^†^** [−0.01, 0.15]	−0.04 [−0.13, 0.06]
Native	0.05 [−0.03, 0.14]	0.04 [−0.06, 0.14]	0.02 [−0.01, 0.05]	0.00 [−0.03, 0.04]	0.23*** [0.06, 0.41]	0.09 [−0.11, 0.29]	0.12* [0.00, 0.23]	0.07 [−0.06, 0.21]
Asian and Pacific Islander	−0.14** [−0.26, −0.02]	−0.18** [−0.32, −0.04]	0.00 [−0.04, 0.05]	−0.02 [−0.06, 0.02]	0.01 [−0.24, 0.26]	−0.20 [−0.47, 0.06]	−0.20** [−0.37, −0.04]	−0.25*** [−0.43, −0.07]
Other	−0.13*** [−0.21, −0.06]	−0.13*** [−0.22, −0.04]	0.02 [−0.00, 0.05]	0.00 [−0.03, 0.03]	0.12 [−0.03, 0.27]	−0.27*** [−0.45, −0.09]	**−0.08^†^** [−0.18, 0.03]	−0.12* [−0.24, −0.00]
N	24,301	18,771	24,301	18,771	24,301	18,771	24,301	18,771

The independent variable in these models is participant race, with each coefficient representing a racial group compared to the weighted grand mean of all other racial groups. The dependent variable in each model is listed in the column titles as the lifetime number of losses, BCL, BLS, or DLS index. The Bonferroni correction adjusted for multiple comparisons.

^†^*P* < 0.10, *P < 0.05, **P < 0.01, ***P < 0.001. Bold indicates a coefficient statistically significant at *P* < 0.10.

For the BCL index, Black participants (β = 0.03, 95% CI [0.02, 0.05]) had higher index scores over the lifetime compared to all other participants in the unadjusted model, while White participants (β = −0.03, 95% CI [−0.04, −0.02]) had lower index scores compared to all other participants. When controlling for covariates, the effects for both Black and White participants remained statistically significant.

For the BLS index, Black (β = 0.24, 95% CI [0.16, 0.31]) and Native (β = 0.23, 95% CI [0.06, 0.41]) participants had higher index scores over the lifetime compared to all other racial groups in the unadjusted model. On the other hand, White participants (β = −0.20, 95% CI [−0.27, −0.14]) had lower index scores compared to all other participants. When controlling for covariates, the significant group differences remained only for Black participants (β = 0.11, 95% CI [0.02, 0.20]). Additional effects emerged for Hispanic (β = −0.17, 95% CI [−0.31, −0.03]) and “Other” (β = −0.27, 95% CI [−0.45, −0.09]) participants who had lower scores compared to all other participants.

For the DLS index, Black (β = 0.15, 95% CI [0.10, 0.20]) and Native (β = 0.12, 95% CI [0.00, 0.23]) participants had higher index scores over the lifetime compared to all other participants, while White (β = −0.11, 95% CI [−0.15, −0.07]) and Asian and Pacific Islander (β = −0.20, 95% CI [−0.37, −0.04]) participants had lower index scores in the unadjusted model. The effects remained significant for Black, Asian, and Pacific Islander participants and marginally significant for White participants after controlling for covariates. Notably, sensitivity analyses that calculated the DLS index restricted to losses that had a confirmed exact age of deceased at death yielded similar results (*SI Appendix*, Table S4).

Racial differences were less detectable when examining the number of losses. Asian and Pacific Islander (β = −0.14, 95% CI [0-0.26, −0.02]) and “Other” (β = −0.13, 95% CI [−0.21, −0.06]) participants had fewer losses over the lifetime than all other racial groups in the unadjusted model. When controlling for covariates, these effects remained statistically significant, and an additional effect emerged for Black participants who had more losses over the lifetime than all other participants (β = 0.05, 95% CI [0.00, 0.10]).

When examining racial differences in loss indices for specific type of relationship only (i.e., parent losses only), Black participants experienced a significantly higher burden of loss for child and sibling losses (i.e., family members in the same or younger generation), and had a significantly lower burden of loss for parent and spouse losses (i.e., family members in the same or older generation) (*SI Appendix*, Table S5). We also ran sensitivity analyses including participants with no recorded losses, finding that effects among Black participants and Native participants remained but were attenuated in all unadjusted models and in adjusted models for the BCL and DLS indices; the only finding that became nonsignificant was the greater burden of loss for Black participants in the adjusted model for the BLS index (*SI Appendix*, Table S6). Finally, findings remained significant in sensitivity analyses specifying a Poisson regression to account for the number of losses and BCL indices as count outcomes (*SI Appendix*, Table S7).

### Aim 2: Do baseline loss index scores predict all-cause mortality?

**Fig. 3.**
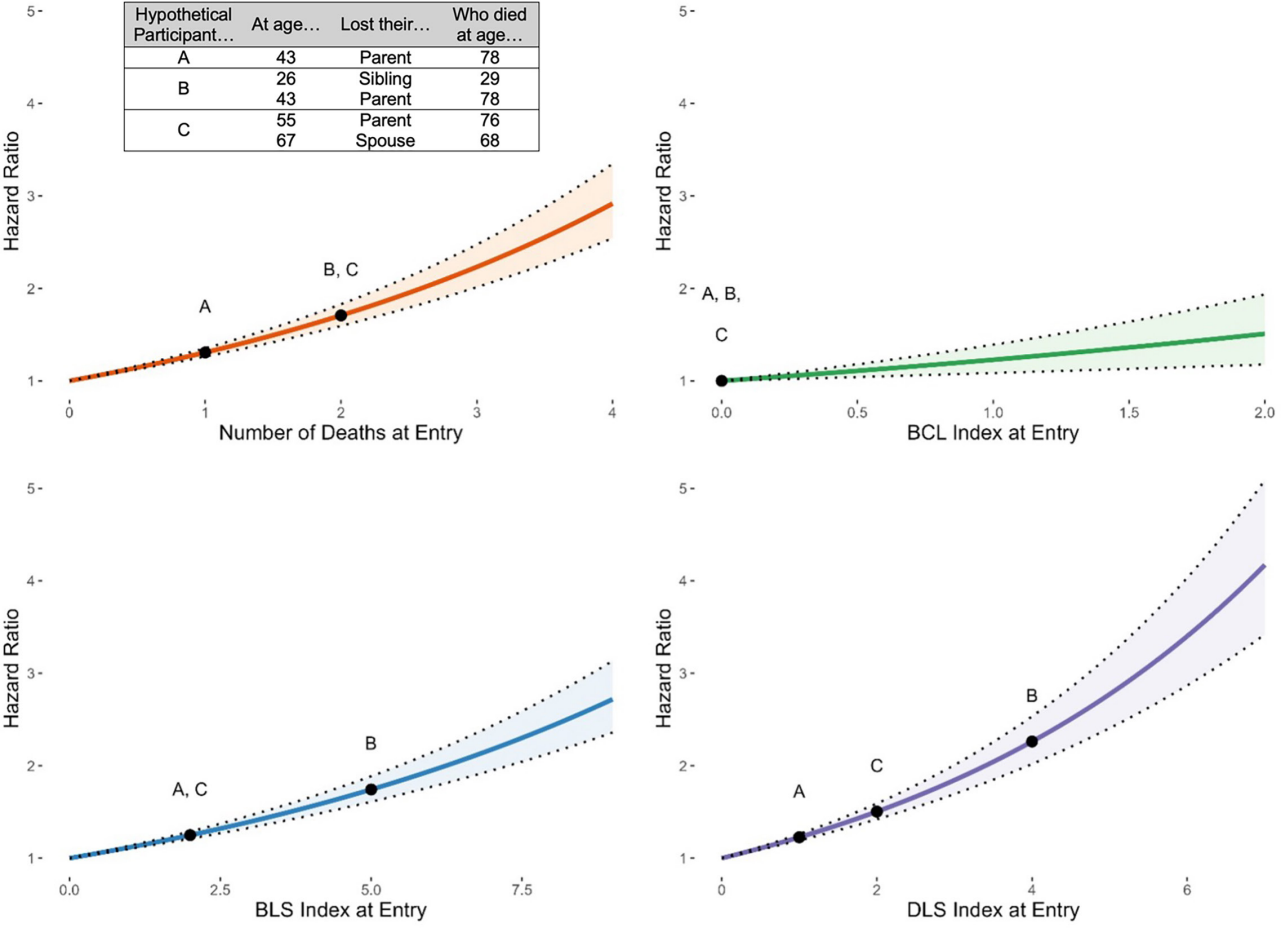
Visual depiction of hazard ratios functions and 95% CI listed in *SI Appendix*, Table S6, “Total Sample,” for adjusted models with loss index at entry predicting all-cause mortality during the study period controlling for covariates. To help illustrate differences in effect sizes based on possible losses that a given individual might have, we constructed a legend of hypothetical participants and their corresponding losses, ages of bereavement, and ages of deceased loved one dying. This legend is meant to show how the same loss(es) could result in differently calculated scores across each of the indices (x-axis), and a corresponding different odds of mortality (y-axis).

During the study period, 30.68% of participants died. All-cause mortality results are displayed in Table 3. Higher loss index scores at study enrollment predicted higher odds of all-cause mortality during the study period across all three indices and the number of losses in both unadjusted and adjusted models, with effects attenuating after controlling for sociodemographic and health covariates. Fig. 3 visualizes the hazard ratios based on adjusted models for each index on the y-axis, and the range of scores observed for each index on the x-axis. For example, the hazard function appears flattest for the BCL index and steepest for the DLS index. The legend also provides hypothetical examples of participants’ losses, and the figure illustrates their corresponding loss index scores and odds of mortality during the study period. Notably, the BCL index was least able to characterize the differential impact of loss burden on mortality.

**Table 3.** Cox mixed-effects results with loss index at study entry predicting all-cause mortality during the study period

	Number of Losses		Bereaved-dependent childhood losses (BCL) index		Bereaved-dependent life stage (BLS) index		Deceased-dependent life stage (DLS) index	
	Unadjusted	Adjusted	Unadjusted	Adjusted	Unadjusted	Adjusted	Unadjusted	Adjusted
Total sample	1.31*** [1.28, 1.35]	1.31*** [1.26, 1.35]	1.42*** [1.30, 1.55]	1.23*** [1.08, 1.39]	1.14*** [1.13, 1.16]	1.12*** [1.10, 1.14]	1.25*** [1.22, 1.28]	1.23*** [1.19, 1.26]
Race-stratified								
White	1.32*** [1.28, 1.37]	1.30*** [1.25, 1.35]	1.41*** [1.27, 1.57]	1.18* [1.01, 1.37]	1.14*** [1.13, 1.16]	1.12*** [1.10, 1.14]	1.27*** [1.24, 1.31]	1.24*** [1.20, 1.28]
Black	1.35*** [1.26, 1.45]	1.33*** [1.21, 1.46]	1.42*** [1.19, 1.70]	1.42* [1.05, 1.90]	1.14*** [1.11, 1.18]	1.13*** [1.08, 1.18]	1.22*** [1.17, 1.29]	1.21*** [1.13, 1.31]
Hispanic	1.11 [0.97, 1.27]	1.05 [0.99, 1.25]	0.89 [0.60, 1.34]	0.81 [0.48, 1.37]	1.04 [0.98, 1.10]	1.00 [0.93, 1.07]	1.03 [0.94, 1.13]	0.98 [0.87, 1.11]
Native	**1.18^†^** [0.99, 1.40]	**1.25**^†^**** [0.98, 1.61]	**1.45^†^** [0.95, 2.21]	1.63 [0.72, 3.67]	1.09* [1.01, 1.18]	1.08 [0.97, 1.21]	1.15* [1.00, 1.32]	1.24* [1.02, 1.50]
Asian and Pacific Islander	1.63* [1.06, 2.50]	1.36 [0.85, 2.18]	2.87* [1.12, 7.36]	3.27* [1.07, 9.97]	1.24* [1.03, 1.47]	1.18* [0.97, 1.44]	1.46* [1.05, 2.03]	1.32 [0.92, 1.89]
Other	1.27* [1.02, 1.57]	1.73** [1.24, 2.43]	0.96 [0.48, 1.91]	1.30 [0.43, 3.98]	1.08 [0.98, 1.19]	1.22* [1.04, 1.43]	1.11 [0.96, 1.28]	1.49** [1.14, 1.95]

^†^*P* < 0.10, *P < 0.05, **P < 0.01, ***P < 0.001. Bold indicates a coefficient statistically significant at *P* < 0.10.

Findings held across unadjusted and adjusted models in the race-stratified results for White participants and Black participants, who had the largest samples (Table 3). Effects also remained for parent and spouse losses (*SI Appendix*, Table S8) that were the most commonly occurring losses in this sample, for sensitivity analyses including participants with no recorded losses except in the case of the BCL adjusted model (*SI Appendix*, Table S9), and for sensitivity analyses that restricted the DLS index to only losses with the deceased’s exact age at death (*SI Appendix*, Table S10).

## Discussion

The present findings simultaneously quantify racial inequities in both premature and cumulative loss burden. Across indices, Black participants were the racial group who had the highest loss index scores, consistent with prior work (5, 51). Across unadjusted models for the BLS and DLS indices, Native participants also consistently had a higher burden of loss than other participants. Regarding racial life expectancy gaps, the Native American population has the lowest life expectancy of all US racial groups (52). Despite this distressing evidence, no work on inequities in loss and grief had previously been conducted among Native Americans. Given recent crises of loss from COVID (53) and police brutality that the most recent wave of data collected in 2020 does not fully represent, racial disparities in loss are likely even more pronounced and relevant in the present moment. For example, the estimated racial life expectancy gap for Native Americans widened due to COVID, from living 4.5 y fewer than White Americans in 2020 to living 6.4 y fewer in 2022 (54).

Results in Hispanic participants showed nuanced results—compared to all other participants, they had lower lifetime loss burden on the BLS index after controlling for covariates and marginally higher lifetime loss burden on the DLS index without controlling for covariates. In other words, Hispanic participants were less likely to have lost family members when they themselves were younger in age, but still experienced more losses of family members who died before their projected life expectancy. This finding is partially consistent with the “Hispanic health paradox” at the national level that shows lower rates of major causes of death and longer life expectancies compared to White Americans due to factors including sociocultural processes (e.g., *simpatía*) and social cohesion in intergenerational households that provide health advantages (55). However, greater acculturation and longer time spent in the United States also confer worse health outcomes in this population (55), and our results may reflect the acculturation status in participants’ kin networks. Asian and Pacific Islander participants overall had a lower burden of loss on the DLS index compared to White participants, which may be partially attributed to factors such as higher educational attainment that may capture access to healthcare, diet, job security, and health literacy (56). However, these effects should be interpreted with caution due to the aggregation of Asian ethnic groups that overlooks disparities in life expectancy among Pacific Islanders (57), South Asians, and Southeast Asians (56, 58), as well as the aggregation of Hispanic participants across nativity status (59). “Other” participants additionally had lower lifetime loss burden on the BLS and DLS indices, but the lack of specificity regarding racial category composition precludes drawing clear conclusions. Finally, when examining specific relational losses (i.e., parent losses only), Black participants had a higher burden of loss for child and sibling losses compared to all other participants. Yet for spousal and parental loss, Black participants had a lower burden of loss while White participants had a higher burden of loss. The finding for spousal loss may possibly be explained by older age at marriage and lower marriage rates among Black individuals compared to White individuals (60), while the finding for parental loss may be partially explained by greater prevalence of single-parent households and family separation among Black families, including from incarceration, family policing, and poverty (61–63).

This study also prospectively relates the combined contribution of both premature and cumulative loss burden to higher mortality risk among communities of color. Greater occurrence and prematurity of loss related to higher all-cause mortality rates across all indices, ranging from 12 to 92% higher odds during the study period for each additional loss. Additionally, our race-stratified results corroborated that this relationship held among the racial group with the largest sample size among participants of color (Black participants). Other racial groups such as Native participants inconsistently showed differences in race-stratified models (i.e., BLS and DLS but not BCL indices), possibly due to smaller sample sizes. The different quantifications of prematurity produced similar results for predicting all-cause mortality, though this relationship was strongest when measured with the DLS index. A major implication of this work is that the relationship between premature exposure to death and premature mortality may point toward persistent cycles of premature death in Black and Native American communities.

### Comparing All Three Indices of Lifetime Loss.

Our work evaluated three different indices: a Bereaved-dependent Childhood Losses index that counts only losses occurring earliest in the participant’s life, a Bereaved-dependent Life Stage index that counts and weighs all losses based on the developmental stage of participant exposure to loss, and a Deceased-dependent Life Stage index that counts and weighs losses based on the developmental stage of the deceased’s age at death.

In our view, the BCL approach is least recommended for modeling lifespan loss and health. This index did not incorporate spouse and child losses, which are highly unlikely to occur by the age of 17. Losses that occur later in life are not summed in this index, such that *quantity* of lifetime loss is not well-represented. However, the BCL index may be especially valuable for researchers interested in outcomes in emerging adulthood, such as educational attainment and family formation. The other two indices may be more useful for researchers modeling midlife and later-life outcomes.

To quantify prematurity, we recommend calculating lifetime loss using the BLS approach when the bereaved’s age of exposure to loss is available. This method captures ordinal differences in prematurity of loss exposure and counts all losses, even later in life.

The DLS approach accounts for relevant characteristics of the deceased and uses an intuitive definition of premature death as the deceased dying at a young age. The DLS index shares the benefits of the BLS index in measuring quantity of loss and in variation in prematurity as an ordinal variable. The data required for calculating this index is also not burdensome—deceased’s age at death. This index had the strongest relationship to all-cause mortality out of all indices tested. However, the DLS index’s focus on characteristics of the deceased means that it may be less informative in predicting other outcomes in the bereaved, such as associations with other lifetime stressors.

Finally, compared to the BCL index (i.e., prematurity only) and the number of losses (i.e., quantity only), the BLS and DLS indices more consistently revealed greater burdens of loss in Black and Native American participants for Aim 1, suggesting that the combined measurement of prematurity and quantity of loss is more useful in characterizing racial disparities in loss. For Aim 2, all three tested indices as well as the number of losses were useful in predicting all-cause mortality, though the DLS index overall showed the largest effect sizes.

### Future Directions and Implications.

Across both Aims 1 and 2, several limitations emerge in the framing of race, sample characteristics, and measurement approaches, all of which suggest important future directions for research. Regarding race, White participants comprised nearly 68% of the sample, which is comparable to the proportion of non-Hispanic White Americans in national Census averages for older adults over 55 y old. While the racial makeup of this sample was critical for population-based estimates, future research should devote resources toward studying loss specifically in marginalized groups, who are inherently worthy of study. This work can also be extended to highlight the legacies of resistance and collective ritual that these communities have engaged in even amid loss, which remain unexplored in this study. The conceptualization of race in HRS was also limited by broad categories that excluded racial groups such as Southwest Asian/North African participants and multiracial participants who were likely grouped together under the “Other” racial category. HRS also only assessed ethnicity among participants who endorsed Hispanic identities, thus neglecting potential nuances in findings by ethnicity. Future research on the differential burden of loss must consider these groups.

Sample characteristics limited generalizability in several ways. We excluded a significant portion of the sample due to potential missingness in recorded losses that we could not verify. Most excluded participants (42.6%) were from the AHEAD cohort, which is the oldest cohort of HRS that has near-complete mortality follow-up, whereas few included participants (5.7%) were from the AHEAD cohort. Because older people are more likely to have experienced more losses than younger people, it was unusual that those in our sample with no kin losses reported were significantly older than those with at least 1 kin loss reported. Sensitivity analyses including participants with no losses revealed that results remained significant across both aims, except in the case of racial disparities measured by the BLS index after controlling for covariates and in mortality risk measured by the BCL index after controlling for covariates. As our included sample is no longer nationally representative, results should be interpreted with caution and not generalized as an epidemiological estimate of loss burden in the United States. The sample consisted of older adults over 50 y old, and although we controlled for age in our adjusted analyses, results do not reflect other possible generational differences in loss burden unique to younger individuals in the United States, such as access to publicly and highly visible losses by COVID and police brutality through technology and social media. Our results are specific to those who have already survived until older adulthood and participated in a study spanning multiple waves of data collection, so this sample may reflect advantages across socioeconomic status and access to resources. Findings likely underestimate racial disparities in loss burden due to the exclusion of most disadvantaged individuals who died before reaching age 50.

Several features of measurement limit the capacity of our indices to characterize loss burden and interpretation of the results. First, the focus on immediate kin loss in the present study introduced potential confounding due to family structures, degree of contact, and race of family members. Results are likely an underestimate of the burden of loss faced by participants, as sibling and child deaths are underreported in HRS. The loss of an important aunt, grandparent, or friend was not measured in the present study. Moreover, Fig. 1 suggests a strong relationship between the BLS and DLS indices—that when the bereaved experienced loss at a younger age, the deceased may have also died prematurely. Thus, several plausible explanations for the all-cause mortality results remain: 1) that loss burden predicts earlier death among the bereaved, and 2) that the same factors contribute to earlier death among both the deceased loved ones and the bereaved who are left grieving their deaths. Genetic factors contributing to the deceased’s death may directly influence the health of biologically related bereaved, such as shared genetic factors influencing impulsivity, psychopathology, and substance use (44). Genetic research specific to family member bereavement is presently limited (44) but unlikely to fully explain present findings, which show increased mortality risk after the loss of spouses, who are not biologically related to the bereaved.

Second, we weighted each type of relational loss (i.e., mother, father, sibling, child, spouse) equally in our indices. In the absence of relevant data on participants’ level of closeness to deceased or severity of grief after death, we were unable to implement more sophisticated weighting procedures. Weighting a sibling loss as universally more burdensome than a parent loss, for example, would be problematic in the absence of empirical data that systematically compared relationships to deceased and concluded which losses are most burdensome. Future research may benefit from assessments of relational closeness and grief severity to weight the losses of the closest loved ones as most burdensome. The only data related to losses that exist in HRS were age—specifically, age of bereaved at loss and age of deceased at death—which are not typically available in bereavement studies. Weighting losses by years of age was unintuitive and difficult to interpret (See *SI Appendix*, *Methods*, Loss Index Development, DTL continuous index). We thus weighted losses by developmental stage of exposure or of death for an intuitive approach to calculation that incorporated the dimension of prematurity consistent with developmental frameworks (i.e., the ACEs literature). However, this scaling shares the limitations inherent to ordinal variables. A 1-unit difference in childhood and emerging adulthood loss may not be equal to the 1-unit difference in middle adulthood and late adulthood loss.

Third, the low observed correlations between our loss indices and lifetime trauma, recent stressors, and chronic stressors suggest that loss may be a distinct type of stressor compared to other stressful life events. At the same time, more proximal, less dramatic, and enduring stressors may have differing psychological and material impacts on people compared to significant life events such as loss (64). Without additional context on the types and duration of stressors participants faced, the present study does not provide explanations for how loss and other stressors are differently related to one another. Finally, the structural conditions by which Black and Native participants experience earlier and repeated losses, such as carceral violence and housing insecurity, were not examined in this study. Following in the framework of axes of personal and collective grief (65), identifying the “direct and indirect acts of racist violence” that contribute to the structural antecedents of loss will be critical in disrupting generations of premature loss (65).

A key theme of the present research is understanding premature and cumulative death—and its structural determinants—as a lens for identifying suffering and inequity in marginalized communities. The results in this paper advance the emerging field of “loss and grief disparities,” or inequities in exposure to and impact of loss, in several ways. First, while research is typically limited to the impacts of a single loss at a single life stage, the loss indices we conceptualized and tested have the advantage of parsimony—with one score reflecting several dimensions of loss across longer timescales. Future researchers can employ our indices in their work. These indices directly respond to recent calls from leading experts (31, 66) to move beyond counting losses to help provide future opportunities for researchers to study underlying mechanisms that link event exposures to outcomes. Second, this study also compared various loss measurement approaches against each other, including one based in the popular ACEs measurement. Across both aims, the combined characterization of premature and cumulative loss held the most import. Third, we provide evidence that incurring each additional premature loss of a family member relates to higher odds of mortality during the study period across all three tested indices. Returning to the point that premature death is a beginning point for bereaved communities, this line of work holds implications for how loss may become embedded in health processes, mental health burdens, and decreased access to resources and supports (e.g., healthcare and employment) for the bereaved—thus contributing to cycles of premature grief and premature death. Fourth, our research examines previously understudied groups. We provide evidence of earlier and greater exposure to death in Native American participants, who have among the highest loss burden of all examined racial groups. Last, this work has methodological strengths, as it uses a large, population-based study controlling for sociodemographics, health, and family structure. The study prospectively tracks lifetime loss and outcomes in a naturalistic context across decades, thus making possible nuanced investigations that have not yet been explored. Taken together, learning from those who have experienced losses too soon and too much necessitates using avenues in policy and community organizing to address the structural antecedents of premature death such as lack of healthcare, eviction, and carceral system exposure. It also necessitates identifying interventions at individual-, group-, community-, and policy levels that help bereaved persons meet their health, logistical, and financial needs following loss such as paid bereavement leave and crisis funds. Through these actions, we can work toward more livable futures in which oppressed communities can instead live long, full lives.

## Supplementary Material

Appendix 01 (PDF)

## Data Availability

Some study data are available. The data analyzed in this study originate from the HRS (67). Race/ethnicity variables are categorized as “restricted data,” requiring approval from HRS staff to access within their secure data enclave. While the 73 datasets used to construct the loss index scores are publicly available HRS data, all analyses were conducted within the HRS secure data enclave, preventing data export. Researchers interested in accessing these data may apply for approval through HRS (https://hrs.isr.umich.edu/data-products) (67).

## Footnotes

^*^Controlling for participant death and time in the study allowed us to distinguish between lifetime loss scores based on characteristics of participant attrition. For example, the covariate of participant death by the 2020 wave would be able to account for variation between a hypothetical participant with a lower loss score who died at age 55 and a hypothetical participant with a higher loss score who is still alive at age 75. On the other hand, the covariate of years enrolled in the study would be able to account for variation between two participants who are both still alive or both deceased. For instance, this covariate could distinguish between a hypothetical living participant with a higher loss score who was enrolled in the study for 25 y and another hypothetical living participant with a lower loss score who was enrolled in the study for only 5 y.
