# Supplementary material for: Measuring premature and cumulative family member bereavement: Racial disparities and later mortality risk
Source: Proc Natl Acad Sci U S A. 2025 Jun 10;122(24):e2313600122. doi: 10.1073/pnas.2313600122 (PMC12184363; doi:10.1073/pnas.2313600122)
Supplement: Supplementary file 1 — Appendix 01 (PDF) [file pnas.2313600122.sapp.pdf]

PNAS

Supporting Information for

Measuring Premature and Cumulative Family Member Bereavement: Racial Disparities and Later Mortality Risk

Michelle Chang, M.A., Theodore F. Robles, Ph.D.

Michelle Chang

Email: changmichelle@g.ucla.edu

This PDF file includes:

Supporting text

Tables S1 to S11

SI References

Supporting Information Text

Methods

Loss Index Development

We conceptualized three indices to capture the prematurity and quantity of deaths grieved over the lifespan. Notably, our index scores are likely an underestimate due to the assessment of kin deaths over the lifespan in HRS. Sibling and child death deaths were collected at the household level. Sibling deaths were also not assessed after both parents were reported deceased, and date of sibling death was not assessed at many waves.

While sibling deaths also could have been calculated by identifying decreases in number of living siblings across waves, we chose to report results based on our preregistered analyses (https://osf.io/3j84a). When running analyses that included sibling deaths as decreases in number of living siblings, our effects for Black participants and Native American participants having a greater burden of loss than White participants replicated across all unadjusted and adjusted models and were even of a greater magnitude.

In the process of finalizing these three indices, we tested and preregistered various iterations of conceptualizing loss burden. In our first iteration, we conceptualized the following three indices. All three of these indices produced results for Aim 1 that Black participants and Native American participants had a greater burden of loss over the lifetime compared to White participants across unadjusted and adjusted models, and for Aim 2 that each additional premature loss at study enrollment related to higher odds of mortality during the study period across unadjusted and adjusted models.

Bereaved-dependent Earliest Losses (BEL) index. This index only counts the number of earliest losses. For each type of loss (e.g., loss of a mother), losses occurring in the earliest quartile of age of exposure to that death (0-25th percentile) were coded as 1, and all other losses occurring after the earliest quartile were coded as 0.

Bereaved-dependent Timing of Losses (BTL) index. This index includes all losses grieved over the lifespan, even those that were grieved later in life. For each type of relationship (i.e., mother, son, etc.), the ages of exposure for the 25th, 50th, and 75th percentiles were obtained. Age of exposure to death was assigned a numerical score of 4 if it fell between the 0-25th percentile for that type of loss in the total sample (earliest exposure), 3 if it fell between the 25-50th percentile for that type of loss (earlier exposure), 2 if it fell between the 50-75th percentile for that type of loss (later exposure), or 1 if it fell between the 75-100th percentile for that type of loss (latest exposure). This process was repeated for every recorded death in a participant's lifespan, and a participant's scores were finally summed to account for quantity of losses and produce the final index score for each participant.

Deceased-dependent Timing of Losses (DTL) index. To address limitations of the BTL approach that rarity equates to prematurity and that scores rely on sample characteristics rather than specific definitions of risk, we created another composite measure. Therefore, we conceptualized prematurity of loss in terms of the deceased dying before their projected life expectancy using U.S. life expectancy tables from 1900-2018 produced by the National Center for Health Statistics (1). Whenever available, we utilized data on the deceased's birth year, age at death, and/or year of death when calculating scores. When such data on the deceased were not available, we approximated their projected life expectancy and age at death. A family member death was coded as 2 (premature) if they died before their projected life expectancy, and as 1 if they died at or later than their projected life expectancy.

In response to the limitations we identified that the BEL and BTL indices relied on sample characteristics and thus would produce scores for a given person's losses that would vary from sample to sample, a reviewer proposed weighting losses for the BEL and BTL indices by developmental stage of exposure to loss instead. We thus produced the current BEL and BLS indices listed in the next section (See SI, Loss Index Calculation). The reviewer also raised concerns that the DTL index was not capturing more nuance as a dichotomized variable, so we conceptualized a continuous version of the DTL index.

Deceased-dependent Timing of Losses (DTL) continuous index. We alternatively conceptualized prematurity of loss in terms of the deceased dying before their projected life

expectancy, rather than on characteristics related to the bereaved's age of exposure to death. For this index, we conceptualized prematurity of loss as the deceased dying before their projected life expectancy using U.S. life expectancy tables from 1900-2018 produced by the National Center for Health Statistics (1). Whenever available, we utilized data on the deceased's birth year, age at death, and/or year of death when calculating scores. When such data on the deceased were not available, we approximated their projected life expectancy and age at death. A family member death's prematurity was calculated as the difference between the deceased's estimated or actual age at death and the deceased's projected life expectancy for someone of their sex born in their birth year, to create a continuous measure.

The continuous DTL index again found for Aim 1 that Black participants and Native American participants had a higher burden of loss than White participants, though participants in the HRS sample mostly lost family members who died after their expected life expectancy—producing negative scores on this index. As a result, we identified concerning limitations with the continuous DTL index that it does not capture quantity of losses well because losses could "cancel" each other out. Several examples illustrate this point: A loss with a negative difference between estimated life expectancy and actual age at death can cancel out with a loss with a positive difference when summed together. In another example, experiencing a greater quantity of losses that each have a negative difference in life expectancy and actual age at death is considered a *lower* loss burden than someone who experienced no losses. For Aim 2, the continuous DTL index was not useful in predicting all-cause mortality due to this limitation and the small unit (in years) of this index.

Finally, we conceptualized a simpler version of the deceased-dependent index based upon the same range of values as the BLS index, and that no longer used U.S. life expectancy tables. The current DLS index is described below (See SI, Loss Index Calculation).

Loss Index Calculation

Lifespan developmental theories are embedded within foundational models such as the biopsychosocial model, positing that we can organize age-related patterns of mental, emotional, and physical health as well as their risk and protective factors into developmental contexts that unfold across the lifespan (2, 3). Age-related cutoffs for life stages vary greatly across researchers and are relative approximations, as life events and roles may often be more important in defining these periods (4).

Early life or childhood has received consistent attention in research as a notable developmental period marked by substantial change, from infancy to adolescence. Experiencing loss of a family member in this developmental stage is linked to a variety of short and long-term health outcomes through pathways including dysregulated biological stress response and emotional reactivity (5). The ACEs literature has defined this stage as ages 0-17 to represent the period before reaching adulthood (6). Next, emerging adulthood is relatively newer developmental stage defined as ages 18-29—a time that is neither extended adolescence nor young adulthood (7). This life stage is marked by instability and uncertainty in education, jobs, and social networks while accepting increasing responsibility and independent decision making (7). Losses occurring during emerging adulthood may exacerbate the identity struggles and low social support typical to this period when people serve the fewest roles or obligations to others (7). Moreover, deaths of family members who died in childhood and emerging adulthood also produce lasting adverse effects among the bereaved, who report worse wellbeing, more health problems, relational disruption, and loss of purpose decades later (8).

Middle adulthood is a life stage characterized by the crossroads of increasing stability and wellbeing but decreasing cognitive and physical functions (9). Bereavement during this stage is both subject to the influence of early life histories of loss and predictive of later life outcomes (10). When the deceased die at this stage, the bereaved family members who relied on their caregiving roles and income may experience compounding losses (4). Researchers and the public agree that midlife occurs between 40-60 years old, plus or minus 10 years (4). We selected the age range of 30-50 years old to be closer to the starting age of measurement at 25 years old in the longitudinal Midlife Development in the U.S. (MIDUS) survey, which has been foundational

to the study of this stage (11). Older adulthood, on the other hand, is marked by age-related stresses such as cognitive impairment, loss of work and community roles, and functional limitations, in addition to acquired expertise in life skills and greater focus on meaning in life (12, 13). Bereavement during older adulthood is a more frequent occurrence that reflects increasingly smaller social networks, though the toll of bereavement on older adults depends on various relational and contextual factors (14). Similarly, the deceased dying during this stage is also more common. We selected the age range of 51 years and older to be consistent with past definitions of older adulthood by prevention scientists (2) and the age at entry into the Health and Retirement Study (15, 16), reflecting an age when private pension plans offer strong retirement incentive.

Bereaved-dependent Childhood Losses (BCL) index. This index only counts the number of earliest losses. For each loss, losses occurring in earliest in life—when the bereaved's age of exposure to the loss was between 0 to 17 years old—were coded as 1, while all other losses occurring later in life were coded as 0. We chose to operationalize early life exposure within the 0-17 age range consistent with the childhood trauma conceptualization in HRS measures and with the Adverse Childhood Experiences (ACEs) literature.

Bereaved-dependent Life Stage (BLS) index. This index includes all losses grieved over the lifespan, even those that were grieved later in life. Each loss was weighted by the prematurity of the bereaved's developmental stage during loss exposure. The bereaved's age of exposure to death was assigned a numerical score of 4 if it occurred during ages 0-17 (early life), 3 if it occurred during ages 18-29 (emerging adulthood), 2 if it occurred during ages 30-50 (middle adulthood), or 1 if it occurred after age 50 (older adulthood, baseline enrollment in HRS). This process was repeated for every recorded death in a participant's lifespan, and a participant's scores were finally summed to account for quantity of losses and produce the final index score for each participant.

Deceased-dependent Life Stage (DLS) index. We alternatively conceptualized prematurity of loss in terms of the prematurity of the deceased's age at death, rather than on characteristics related to the bereaved's age of exposure to that death. Each loss of a family member was weighted by the prematurity of the deceased's developmental stage at their death. Using a scale identical to the BLS index's, the deceased's actual or estimated age at death was assigned a numerical score of 4 if it occurred during ages 0-17 (early life), 3 if it occurred during ages 18-29 (emerging adulthood), 2 if it occurred during ages 30-50 (middle adulthood), or 1 if it occurred after age 50 (older adulthood, baseline enrollment in HRS). This process was again repeated for every recorded death in a participant's lifespan, and scores were summed for each participant. Whenever available, we utilized data on the deceased's birth year, age at death, and/or year of death to calculate age at death. When not available, we approximated their age at death.

Spouses had low missingness in ages at death (5.5% missing), while sibling data was mostly missing (94.2% missing for sister, 95.9% missing for brother). For the death of a family member expected to be relatively similar in age to the participant (i.e., spouse, brother, or sister) who did not have available data on the deceased's age at death, that family member's age at death was approximated as the difference between the bereaved's actual age of exposure to that death and the median participant age at the birth of their spouse or sibling.

Parents' ages at death were collected with little missingness (1.2% missing for mother, 1.2% missing for father). For the death of a family member expected to be a generation older in age than the participant (i.e., mother or father) who did not have available data on the deceased's age at death, we approximated parent age at death as equal to the sum of median parent age at participant birth and the bereaved's actual age at exposure to parent death.

Finally, children's ages at death were collected with little missingness (3.7% missing for daughter, 4.3% missing for son). For the death of a family member expected to be a generation younger in age to the participant (i.e., son or daughter) who did not have available data on the deceased's age at death, we approximated child age at death as equal to the difference between the bereaved's age of exposure to child death and the median participant age at child birth. This process was repeated for every recorded death in a participant's lifespan, and a participant's

scores were finally summed to account for quantity of losses and produce the final index score for each participant.

Models

For Aim 1, the predictor variable was participant race, and the outcome variable was lifetime loss index based on all recorded deaths in a participant's life. We computed binary contrasts that compared each racial group to the weighted mean of all other racial groups and adjusted for multiple comparisons using the Bonferroni correction. We did not apply HRS sampling weights based on wave of collection because we made use of data at all possible waves that represented participants' lifetime loss exposure. In following HRS guidance for analyses involving longitudinal data, we did not apply sampling weights because base-year weights do not account for participants entering the sample at different waves, and terminal-year weights do not correct for serious biases due to attrition (17). The adjusted multilevel model is below.

Level 1:

$$lossIndex_{ij} = b_{0i} + b_{1i}race_{ij} + b_{2i}ageBaseline\_cgm_{ij} + b_{3i}sex_{ij} + b_{4i}SESchildhood_{ij} + b_{5i}SESmotherEducation\_cgm_{ij} + b_{6i}SESfatherEducation\_cgm_{ij} + b_{7i}marriagesBaseline_{ij} + b_{8i}siblingsBaseline_{ij} + b_{9i}childrenEver_{ij} + b_{10i}deceased_{ij} + b_{11i}yearsInStudyBaseline_{ij} + \varepsilon_{ij}$$

Level 2: (clustering variable is household ID):

$$b_{0i} = \beta_{00} + \beta_{01}householdSize\_cgm_j + \beta_{02}liveInSouth_j + \beta_{03}SEScapitalIncome\_cgm_j + \zeta_{0i}$$

$$b_{1i} = \beta_{10}$$

...

$$b_{7i} = \beta_{70}$$

For Aim 2, we tested Cox mixed effects models. The predictor variable was loss index at study enrollment, and the outcome was all-cause mortality. Again, HRS sampling weights based on wave of collection were not applied due to the longitudinal nature of the data. The Cox mixed effects model is below.

Level 1:

$$h_{ij}(t) = h_{0i}(t) * \exp(b_{1i}lossIndex_{ij} + b_{2i}ageBaseline\_cgm_{ij} + b_{3i}sex_{ij} + b_{4i}childhoodSES_{ij} + b_{5i}SESmotherEducation\_cgm_{ij} + b_{6i}SESfatherEducation\_cgm_{ij} + b_{7i}marriagesBaseline_{ij} + b_{8i}siblingsBaseline_{ij} + b_{9i}childrenEver_{ij} + b_{8i}smokerBaseline_{ij} + b_{9i}drinkerBaseline_{ij} + b_{10i}chronicConditionsBaseline_{ij} + b_{11i}generalHealthBaseline_{ij} + \varepsilon_{ij})$$

Level 2 (clustering variable is household ID):

$$b_{0i} = \beta_{00} + \beta_{01}householdSizeBaseline\_cgm_j + \beta_{02}liveInSouthBaseline_j + \zeta_{0i}$$

$$b_{1i} = \beta_{10}$$

$\dots$

$$b_{11i} = \beta_{110}$$

Table S1. Descriptive statistics for all variables across excluded and analytic samples.

Variable	Excluded Sample (N = 14,404) %	Analytic Sample (N = 27,985) %	X^2(df), p-value (test of indep)	Effect size (Cramer's V)
Race			135.65(5) *p*<.0001	0.06
White American	71.94%	67.88%		
Black / African American	17.39%	17.81%		
Hispanic American	5.57%	6.53%		
Native American / Alaska Native / Native Hawaiian	1.70%	2.60%		
Asian American / Pacific Islander	1.25%	1.40%		
Other	2.16%	3.77%		
	N = 13,132	N = 24,301		
Sex			49.88(1), *p*<.0001	0.03
Female	53.66%	57.26%		
Male	46.35%	42.74%		
Birth cohort			10,710.96(7), *p*<.0001	0.50
AHEAD born before 1923 as reference)	42.83%	5.65%		
CODA (1924-1930)	7.54%	11.27%		
HRS (1931-1941)	27.42%	23.43%		
War Babies (1942-1947)	3.35%	11.52%		
Early Baby Boomers (1948-1953)	3.15%	15.83%		
Mid Baby Boomers (1954-1959)	4.98%	16.20%		
Late Baby Boomers (1960-1965)	7.56%	13.54%		
No Cohort Reported	3.17%	2.57%		
Childhood family SES			38.96(3), *p*<.0001	0.03
Pretty well off financially	7.30%	8.21%		
About average	58.70%	60.96%		
It varied	1.01%	0.88%		
Poor	32.99%	29.95%		
	N = 10,655	N = 27,568		
Living in South at baseline	42.01%	41.79%	0.19(1) *p*=.66	0.002
	N = 13,855	N = 27,348		
	**Mean (SD)**	**Mean (SD)**	**Effect size (d)**	
Mother's years education	8.87 (3.17)	9.83 (3.80)	0.26	
	N = 12,441	N = 25,179		
Father's years education	8.74 (3.36)	9.56 (4.12)	0.21	
	N = 11,961	N = 23,081		
Household capital income at baseline	$7,550.09 ($91,468.27)	$12,316.25 ($73,956.05)	0.06	
	N = 13,879	N = 27,437		
Household size at baseline	2.29 (1.29)	2.65 (1.35)	0.27	
	N = 13,879	N = 27,437		
Number of times married at baseline	1.29 (0.71)	1.33 (0.76)	0.05	
	N = 13,814	N = 27,354		
Number of children ever born	2.68 (2.12)	2.62 (1.88)	0.03	
	N = 13,181	N = 27,730		
Number of living siblings at baseline	2.56 (2.33)	3.26 (2.61)	0.28	
	N = 13,172	N = 27,325		
Age at baseline	66.26 (12.04)	57.42 (7.46)	0.96	
	N = 13,879	N = 27,437		
Deceased by 2020	64.98% (47.71%)	30.68% (46.12%)	0.74	
	N = 14,404	N = 27,985		
Number of years in study	12.19 (9.22)	14.99 (9.28)	0.33	
	N = 13,880	N = 27,441		

Table S2. Psychometric properties of DLS index inclusive of losses with estimated age at death and DLS index restricted to losses with exact age at death.

		Deceased-dependent Life Stage (DLS) index - estimate	Deceased-dependent Life Stage (DLS) index - exact
All Losses	*M*	1.96	1.86
	SD	1.07	0.95
	Median	2	2
	N	27,985	25,894
Parent Losses	*M*	1.48	1.49
	SD	0.82	0.81
	Median	2	2
	N	27,985	25,894
Spouse Losses	*M*	0.21	0.22
	SD	0.41	0.42
	Median	0	0
	N	27,985	25,894
Child Losses	*M*	0.17	0.18
	SD	0.67	0.67
	Median	0	0
	N	27,985	25,894
Sibling Losses	*M*	0.08	0.00
	SD	0.45	0.07
	Median	0	0
	N	27,985	25,894

Table S3. Pearson correlations between lifetime loss indices and lifetime and recent stressors.

Lifetime Loss Indices	Lifetime Trauma (excluding child death)	Lifetime Trauma	Childhood Trauma	Stressors in Past 5 Years	Chronic Stress Past 12 Months
Number of Losses	**.03*****	**.07*****	-.01	**-.03*****	.00
Bereaved-dependent Childhood Losses (BCL)	**.03*****	**.03*****	**.03*****	**.03*****	.01
Bereaved-dependent Life Stage (BLS)	**.06*****	**.06*****	**.05*****	**.05*****	-.00
Deceased-dependent Life Stage (DLS)	**.05*****	**.10*****	.00	-.01	**.02***

† *p*<.10, **p*<.05, ***p*< .01, ****p*< .001. Bold indicates a coefficient statistically significant at *p*< 0.10.

Table S4. Binary contrasts for multilevel models showing racial disparities in lifetime loss for DLS index inclusive of losses with estimated age at death and DLS index restricted to losses with exact age at death.

	Deceased-dependent Life Stage (DLS) index – estimate		Deceased-dependent Life Stage (DLS) index – exact	
Racial Group	**Unadjusted**	**Unadjusted**	**Unadjusted**	**Adjusted**
Reference: Weighted grand mean of other groups				
White	**-0.11***** [-0.15, -0.07]	**-0.04†** [-0.10, 0.01]	**-0.03*** [-0.07, 0.00]	0.01 [-0.04, 0.06]
Black	**0.15***** [0.10, 0.20]	**0.12***** [0.06, 0.18]	**0.07**** [0.02, 0.11]	**0.06*** [0.00, 0.11]
Hispanic	**0.07†** [-0.01, 0.15]	-0.04 [-0.13, 0.06]	-0.01 [-0.08, 0.06]	**-0.08*** [-0.17, -0.00]
Native	**0.12*** [0.00, 0.23]	0.07 [-0.06, 0.21]	**0.11*** [0.00, 0.22]	**0.09†** [-0.03, 0.21]
Asian & Pacific Islander	**-0.20**** [-0.37, -0.04]	**-0.25***** [-0.43, -0.07]	**-0.17**** [-0.32, -0.02]	**-0.21**** [-0.37, -0.05]
Other	**-0.08†** [-0.18, 0.03]	**-0.12*** [-0.24, -0.00]	-0.08 [-0.17, 0.01]	**-0.13**** [-0.24, -0.02]
N	24,296	18,918	22,275	17,480

† p<.10, *p<.05, **p< .01, ***p< .001. Bold indicates a coefficient statistically significant at p< 0.10.

Table S5. Binary contrasts for multilevel models in which race predicted lifetime loss index scores, stratified by relationship to deceased.

		**Number of Losses**		**Bereaved-dependent Childhood Losses (BCL) index**		**Bereaved-dependent Life Stage (BLS) index**		**Deceased-dependent Life Stage (DLS) index - estimated**		**Deceased-dependent Life Stage (DLS) index - exact**	
		**Unadjusted**	**Adjusted**	**Unadjusted**	**Adjusted**	**Unadjusted**	**Adjusted**	**Unadjusted**	**Adjusted**	**Unadjusted**	**Adjusted**
	Racial Group Reference: Weighted grand mean of other groups										
All Losses	White	-0.00 [-0.03, 0.03]	0.01 [-0.03, 0.05]	**-0.03***** [-0.04, -0.02]	**-0.01*** [-0.02, -0.00]	**-0.20***** [-0.27, -0.14]	0.02 [-0.06, 0.10]	**-0.11***** [-0.15, -0.07]	**-0.04†** [-0.10, 0.01]	**-0.03*** [-0.07, 0.00]	0.01 [-0.04, 0.06]
	Black	0.03 [-0.01, 0.07]	**0.05*** [0.00, 0.10]	**0.03***** [0.02, 0.05]	**0.02**** [0.00, 0.03]	**0.24***** [0.16, 0.31]	**0.11**** [0.02, 0.20]	**0.15***** [0.10, 0.20]	**0.12***** [0.06, 0.18]	**0.07**** [0.02, 0.11]	**0.06*** [0.00, 0.11]
	Hispanic	0.00 [-0.05, 0.06]	-0.05 [-0.12, 0.02]	0.00 [-0.02, 0.02]	-0.00 [-0.02, 0.02]	-0.01 [-0.13, 0.10]	**-0.17**** [-0.31, -0.03]	**0.07†** [-0.01, 0.15]	-0.04 [-0.13, 0.06]	-0.01 [-0.08, 0.06]	**-0.08*** [-0.17, -0.00]
	Native	0.05 [-0.03, 0.14]	0.04 [-0.06, 0.14]	0.02 [-0.01, 0.05]	0.00 [-0.03, 0.04]	**0.23***** [0.06, 0.41]	0.09 [-0.11, 0.29]	**0.12*** [0.00, 0.23]	0.07 [-0.06, 0.21]	**0.11*** [0.00, 0.22]	**0.09†** [-0.03, 0.21]
	Asian & Pacific Islander	**-0.14**** [-0.26, -0.02]	**-0.18**** [-0.32, -0.04]	0.00 [-0.04, 0.05]	-0.02 [-0.06, 0.02]	0.01 [-0.24, 0.26]	-0.20s [-0.47, 0.06]	**-0.20**** [-0.37, -0.04]	**-0.25***** [-0.43, -0.07]	**-0.17**** [-0.32, -0.02]	**-0.21**** [-0.37, -0.05]
	Other	**-0.13***** [-0.21, -0.06]	**-0.13***** [-0.22, -0.04]	0.02 [-0.00, 0.05]	0.00 [-0.03, 0.03]	0.12 [-0.03, 0.27]	**-0.27***** [-0.45, -0.09]	**-0.08†** [-0.18, 0.03]	**-0.12*** [-0.24, -0.00]	-0.08 [-0.17, 0.01]	**-0.13**** [-0.24, -0.02]
Parent Losses Only	White	-0.02 [-0.04, 0.01]	**0.08***** [0.04, 0.11]	**-0.03***** [-0.04, -0.02]	**-0.01*** [-0.02, -0.00]	**-0.19***** [-0.26, -0.13]	**0.09**** [0.01, 0.17]	**-0.05***** [-0.09, -0.02]	**0.05**** [0.01, 0.09]	**-0.05***** [-0.08, -0.02]	**0.04*** [0.00, 0.08]
	Black	-0.01 [-0.04, 0.03]	**-0.07***** [-0.11, -0.03]	**0.03***** [0.02, 0.04]	**0.02**** [0.00, 0.03]	**0.18***** [0.10, 0.26]	-0.02 [-0.11, 0.07]	**0.03*** [0.00, 0.07]	**-0.05*** [-0.09, -0.00]	**0.03†** [-0.01, 0.07]	**-0.04†** [-0.08, 0.01]
	Hispanic	-0.02 [-0.07, 0.03]	-0.04 [-0.10, 0.03]	-0.00 [-0.02, 0.02]	-0.00 [-0.03, 0.02]	-0.06 [-0.18, 0.06]	**-0.17**** [-0.30, -0.03]	-0.02 [-0.08, 0.04]	-0.03 [-0.09, 0.04]	-0.03 [-0.09, 0.04]	-0.02 [-0.09, 0.05]
	Native	0.05 [-0.03, 0.13]	-0.01 [-0.10, 0.07]	**0.02*** [0.00, 0.05]	0.01 [-0.02, 0.04]	**0.21**** [0.03, 0.39]	0.04 [-0.16, 0.23]	**0.09*** [0.00, 0.18]	0.02 [-0.08, 0.12]	**0.10**** [0.01, 0.18]	0.02 [-0.07, 0.12]
	Asian & Pacific Islander	0.06 [-0.05, 0.17]	-0.04 [-0.16, 0.07]	0.01 [-0.04, 0.05]	-0.02 [-0.05, 0.02]	**0.22†** [-0.04, 0.47]	-0.05 [-0.30, 0.21]	0.06 [-0.07, 0.19]	-0.06 [-0.19, 0.07]	0.03 [-0.09, 0.16]	**-0.10†** [-0.23, 0.03]
	Other	**0.10***** [0.04, 0.17]	**-0.07†** [-0.15, 0.01]	**0.02*** [0.00, 0.05]	0.00 [-0.02, 0.03]	**0.32***** [0.17, 0.48]	**-0.20**** [-0.37, -0.03]	**0.14***** [0.07, 0.22]	-0.05 [-0.13, 0.04]	**0.15***** [0.07, 0.22]	-0.05 [-0.14, 0.04]
Spouse Losses Only	White	**0.10***** [0.09, 0.12]	**0.03**** [0.02, 0.05]	N/A		**0.10***** [0.09, 0.12]	**0.03**** [0.01, 0.05]	**0.10***** [0.09, 0.12]	**0.03**** [0.01, 0.05]	**0.10***** [0.08, 0.11]	**0.03**** [0.00, 0.05]
	Black	**-0.08***** [-0.10, -0.06]	**-0.02†** [-0.04, 0.00]			**-0.08***** [-0.10, -0.06]	**-0.02†** [-0.04, 0.00]	**-0.08***** [-0.10, -0.06]	**-0.02†** [-0.04, 0.00]	**-0.07***** [-0.09, -0.05]	-0.01 [-0.04, 0.01]
	Hispanic	**-0.04***** [-0.07, -0.01]	**-0.03*** [-0.07, -0.00]			**-0.04***** [-0.07, -0.01]	**-0.04*** [-0.07, -0.00]	**-0.04***** [-0.07, -0.01]	**-0.03†** [-0.07, 0.00]	**-0.03*** [-0.06, -0.00]	**-0.03†** [-0.07, 0.00]
	Native	**-0.04†** [-0.08, 0.01]	0.02 [-0.03, 0.07]			**-0.03†** [-0.08, 0.02]	0.03 [-0.02, 0.08]	**-0.03†** [-0.08, 0.01]	0.02 [-0.03, 0.07]	**-0.04†** [-0.09, 0.01]	0.02 [-0.03, 0.07]
	Asian & Pacific Islander	**-0.08**** [-0.14, -0.01]	-0.03 [-0.09, 0.03]			**-0.07**** [-0.14, -0.01]	-0.03 [-0.10, 0.04]	-0.08 [-0.14, -0.02]	-0.03 [-0.09, 0.03]	**-0.09***** [-0.15, -0.02]	-0.04 [-0.10, 0.03]
	Other	**-0.17***** [-0.21, -0.13]	**-0.03†** [-0.08, 0.01]			**-0.17***** [-0.21, -0.13]	**-0.04†** [-0.08, 0.01]	**-0.18***** [-0.21, -0.13]	**-0.04†** [-0.08, 0.01]	**-0.18***** [-0.22, -0.14]	-0.04 [-0.09, 0.00]

Child Losses Only	White	**-0.05*****	**-0.05*****	N/A		**-0.05*****	**-0.05*****	**-0.09*****	**-0.06*****	**-0.10*****	**-0.07*****
		[-0.06, -0.03]	[-0.07, -0.03]			[-0.06, -0.03]	[-0.07, -0.03]	[-0.12, -0.06]	[-0.10, -0.03]	[-0.13, -0.07]	[-0.11, -0.04]
	Black	**0.07*****	**0.08*****			**0.07*****	**0.08*****	**0.13*****	**0.12*****	**0.14*****	**0.13*****
		[0.05, 0.09]	[0.06, 0.11]			[0.05, 0.09]	[0.06, 0.11]	[0.09, 0.16]	[0.08, 0.16]	[0.11, 0.18]	[0.09, 0.17]
	Hispanic	0.02	-0.02			0.02	-0.02	**0.05****	-0.02	**0.04†**	-0.03
		[-0.01, 0.05]	[-0.05, 0.02]			[-0.01, 0.04]	[-0.05, 0.01]	[0.00, 0.10]	[-0.08, 0.03]	[-0.01, 0.10]	[-0.09, 0.03]
	Native	0.01	0.01			0.01	0.01	0.02	0.01	0.03	0.03
		[-0.03, 0.05]	[-0.03, 0.05]			[-0.03, 0.05]	[-0.03, 0.05]	[-0.05, 0.09]	[-0.07, 0.08]	[-0.04, 0.10]	[-0.05, 0.10]
	Asian & Pacific Islander	**-0.07****	-0.04			**-0.07****	-0.04	**-0.11****	-0.07	**-0.11****	-0.07
		[-0.13, -0.01]	[-0.10, 0.02]			[-0.13, -0.01]	[-0.10, 0.02]	[-0.21, -0.00]	[-0.17, 0.04]	[-0.22, -0.01]	[-0.18, 0.04]
	Other	**-0.03†**	-0.02			**-0.03***	-0.02	**-0.04†**	-0.04	**-0.04†**	-0.04
		[-0.06, 0.01]	[-0.06, 0.02]			[-0.06, -0.00]	[-0.06, 0.02]	[-0.10, 0.02]	[-0.11, 0.03]	[-0.11, 0.02]	[-0.11, 0.03]
Sibling Losses Only	White	**-0.07*****	**-0.06*****	-0.00	-0.00	**-0.07*****	**-0.05*****	**-0.07*****	**-0.05*****	N/A	
		[-0.08, -0.05]	[-0.07, -0.03]	[-0.00, 0.00]	[-0.00, 0.00]	[-0.09, -0.05]	[-0.08, -0.03]	[-0.09, -0.05]	[-0.08, -0.03]		
	Black	**0.07*****	**0.07*****	0.00	0.00	**0.08*****	**0.07*****	**0.08*****	**0.07*****		
		[0.06, 0.09]	[0.04, 0.09]	[-0.00, 0.00]	[-0.00, 0.00]	[0.05, 0.10]	[0.04, 0.09]	[0.05, 0.09]	[0.04, 0.09]		
	Hispanic	**0.06*****	**0.04****	0.00*	0.00	**0.08*****	**0.06****	**0.08*****	**0.05****		
		[0.03, 0.09]	[0.00, 0.07]	[0.00, 0.01]	[-0.00, 0.01]	[0.04, 0.11]	[0.01, 0.10]	[0.04, 0.11]	[0.01, 0.09]		
	Native	0.03	0.01	-0.00	-0.00	0.03	0.01	0.03	0.01		
		[-0.02, 0.07]	[-0.04, 0.06]	[-0.01, 0.00]	[-0.01, 0.00]	[-0.02, 0.08]	[-0.05, 0.07]	[-0.03, 0.08]	[-0.05, 0.06]		
	Asian & Pacific Islander	-0.05	**-0.06***	-0.00	-0.00	**-0.06†**	**-0.08***	**-0.06†**	**-0.08***		
		[-0.11, 0.01]	[-0.13, -0.00]	[-0.01, 0.00]	[-0.01, 0.00]	[-0.13, 0.01]	[-0.16, -0.00]	[-0.13, 0.01]	[-0.15, -0.00]		
	Other	-0.01	-0.01	-0.00	-0.00	0.01	-0.01	0.00	-0.01		
		[-0.04, 0.03]	[-0.05, 0.04]	[-0.00, 0.00]	[-0.01, 0.00]	[-0.04, 0.05]	[-0.07, 0.04]	[-0.04, 0.04]	[-0.07, 0.04]		
	N	24,301	18,771	24,301	18,771	24,301	18,771	24,301	18,771	24,301	18,771

† $p<.10$, *$p<.05$, **$p<.01$, ***$p<.001$. Bold indicates a coefficient statistically significant at $p<0.10$. Note: BCL results for spouse and child losses are not included because participants did not have deceased spouses or children by age 17. DLS exact results for sibling losses are not included because sibling age at death was not collected by HRS.

Table S6. Binary contrasts for multilevel models showing racial disparities in lifetime loss, for all participants including those with no recorded losses.

	Number of Losses		Bereaved-dependent Childhood Losses (BCL) index		Bereaved-dependent Life Stage (BLS) index		Deceased-dependent Life Stage (DLS) index - estimated		Deceased-dependent Life Stage (DLS) index - exact	
	Unadjusted	Adjusted	Unadjusted	Adjusted	Unadjusted	Adjusted	Unadjusted	Adjusted	Unadjusted	Adjusted
Racial Group Reference: Weighted grand mean of other groups										
White	**-0.08*****	**0.04***	**-0.02*****	**-0.01†**	**-0.25*****	0.05	**-0.15*****	0.01	**-0.08*****	**0.05****
	[-0.11, -0.04]	[0.00, 0.09]	[-0.03, -0.02]	[-0.02, 0.00]	[-0.31, -0.19]	[-0.02, 0.12]	[-0.20, -0.11]	[-0.04, 0.06]	[-0.12, -0.04]	[0.01, 0.10]
Black	**0.04***	0.00	**0.02*****	**0.01****	**0.18*****	0.05	**0.11*****	**0.05***	0.03	-0.01
	[0.00, 0.08]	[-0.05, 0.05]	[0.01, 0.03]	[0.00, 0.02]	[0.11, 0.25]	[-0.04, 0.13]	[0.06, 0.16]	[0.00, 0.11]	[-0.02, 0.08]	[-0.07, 0.05]
Hispanic	**0.08****	**-0.07***	0.00	-0.00	0.10	**-0.18****	**0.12*****	-0.07	0.06	**-0.10****

	[0.01, 0.14]	[-0.15, 0.00]	[-0.01, 0.02]	[-0.02, 0.01]	[-0.02, 0.21]	[-0.31. -0.04]	[0.05, 0.20]	[-0.16, 0.02]	[-0.02, 0.13]	[-0.19, -0.02]
Native	**0.20*****	0.06	**0.02****	0.00	**0.38*****	0.11	**0.25*****	**0.09†**	**0.23*****	**0.09†**
	[0.10, 0.20]	[-0.05, 0.17]	[0.00, 0.04]	[-0.02, 0.03]	[0.22, 0.55]	[-0.08, 0.30]	[0.13, 0.37]	[-0.04, 0.22]	[0.11, 0.33]	[-0.03, 0.22]
Asian & Pacific Islander	-0.05	**-0.20****	0.01	-0.02	0.06	**-0.24***	-0.09	**-0.27*****	-0.06	**-0.22****
	[-0.19, 0.08]	[-0.35, -0.05]	[-0.02, 0.03]	[-0.05, 0.02]	[-0.17, 0.30]	[-0.49, -0.02]	[-0.25, 0.07]	[-0.45, -0.09]	[-0.21, 0.10]	[-0.39, -0.06]
Other	**0.11****	**-0.11*****	**0.03*****	0.00	**0.38*****	**-0.20****	**0.16*****	**-0.11***	**0.15*****	**-0.11***
	[0.02, 0.19]	[-0.21, -0.01]	[0.01, 0.04]	[-0.02, 0.02]	[0.23, 0.53]	[-0.38, -0.03]	[0.06, 0.26]	[-0.23, 0.00]	[0.05, 0.25]	[-0.23, 0.00]
N	37,433	25,963	37,433	25,963	37,433	25,963	37,433	25,963	35,405	24,534

† p<.10, *p<.05, **p< .01, ***p< .001. Bold indicates a coefficient statistically significant at p< 0.10.

Table S7. Binary contrasts for general linear models showing racial disparities in lifetime loss, fitted to Poisson regression.

	Number of Losses		Bereaved-dependent Childhood Losses (BCL) index		Bereaved-dependent Life Stage (BLS) index		Deceased-dependent Life Stage (DLS) index - estimated		Deceased-dependent Life Stage (DLS) index - exact	
	Unadjusted	Adjusted	Unadjusted	Adjusted	Unadjusted	Adjusted	Unadjusted	Adjusted	Unadjusted	Adjusted
Racial Group Reference: Weighted grand mean of other groups										
White	0.00	0.01	**-0.34*****	**-0.16†**	**-0.07*****	0.01	**-0.05*****	-0.02	-0.01	0.01
	[-0.03, 0.04]	[-0.03, 0.05]	[-0.49, -0.21]	[-0.36, 0.03]	[-0.09, -0.04]	[-0.02, 0.04]	[-0.07, -0.02]	[-0.05, 0.02]	[-0.04, 0.02]	[-0.03, 0.05]
Black	0.03	0.02	**0.37*****	**0.26****	**0.08*****	**0.03***	**0.07*****	**0.05****	**0.03†**	0.02
	[-0.03, 0.05]	[-0.02, 0.06]	[0.23, 0.51]	[0.05, 0.47]	[0.05, 0.10]	[0.00, 0.07]	[0.04, 0.10]	[0.01, 0.09]	[-0.00, 0.06]	[-0.02, 0.06]
Hispanic	0.00	-0.03	0.01	-0.01	-0.01	**-0.06****	**0.03†**	-0.02	-0.01	-0.05
	[-0.05, 0.05]	[-0.09, 0.04]	[-0.23, 0.26]	[-0.35, 0.34]	[-0.05, 0.04]	[-0.11, -0.01]	[-0.02, 0.08]	[-0.08, 0.04]	[-0.06, 0.04]	[-0.11, 0.02]
Native	0.03	0.02	**0.28†**	0.10	**0.08*****	0.03	**0.06†**	0.04	**0.06†**	0.05
	[-0.05, 0.11]	[-0.07, 0.11]	[-0.05, 0.56]	[-0.37, 0.57]	[0.02, 0.14]	[-0.04, 0.11]	[-0.02, 0.13]	[-0.04, 0.13]	[-0.02, 0.14]	[-0.04, 0.15]
Asian & Pacific Islander	-0.08	-0.10	0.06	-0.32	0.00	-0.07	**-0.11****	**-0.13***	**-0.10†**	**-0.11†**
	[-0.19, 0.03]	[-0.22, 0.03]	[-0.44, 0.56]	[-1.07, 0.42]	[-0.08, 0.09]	[-0.17, 0.03]	[-0.22, -0.01]	[-0.25, -0.01]	[-0.22, 0.01]	[-0.25, 0.01]
Other	-0.08	-0.08	**0.28†**	0.01	**0.04†**	**-0.10*****	**-0.05†**	**-0.07†**	**-0.05†**	**-0.08†**
	[-0.15, -0.01]	[-0.16, 0.01]	[-0.01, 0.56]	[-0.39, 0.41]	[-0.02, 0.09]	[-0.17, -0.03]	[-0.11, 0.02]	[-0.15, 0.01]	[-0.12, 0.02]	[-0.17, 0.01]
N	24,301	18,771	24,301	18,771	24,301	18,771	24,301	18,771	24,296	17,344

† p<.10, *p<.05, **p< .01, ***p< .001. Bold indicates a coefficient statistically significant at p< 0.10. Note: Models would not converge for specifying mixed effects and Poisson regression, so we simplified the models for this sensitivity analysis by removing the random intercept accounting for participants nested within households.

Table S8. Cox mixed-effects results with loss index for each relationship at study entry predicting all-cause mortality during the study period.

	**Number of Losses**		**Bereaved-dependent Childhood Losses (BCL) index**		**Bereaved-dependent Life Stage (BLS) index**		**Deceased-dependent Life Stage (DLS) index**	
	**Unadjusted**	**Adjusted**	**Unadjusted**	**Adjusted**	**Unadjusted**	**Adjusted**	**Unadjusted**	**Adjusted**
All Losses	**1.31*****	**1.31*****	**1.42*****	**1.23*****	**1.14*****	**1.12*****	**1.25*****	**1.23*****
	[1.28, 1.35]	[1.26, 1.35]	[1.30, 1.55]	[1.08, 1.39]	[1.13, 1.16]	[1.10, 1.14]	[1.22, 1.28]	[1.19, 1.26]
Relationship-stratified								
Parent Only	**1.29*****	**1.31*****	**1.39*****	**1.24*****	**1.13*****	**1.12*****	**1.24*****	**1.26*****
	[1.25, 1.32]	[1.26, 1.35]	[1.27, 1.51]	[1.10, 1.41]	[1.12, 1.15]	[1.10, 1.14]	[1.21, 1.27]	[1.22, 1.30]
Spouse Only	**1.38***	**2.01*****	N/A		**1.42*****	**1.62*****	**1.38***	**1.82*****
	[1.05, 1.82]	[1.48, 2.72]			[1.16, 1.73]	[1.32, 1.98]	[1.08, 1.76]	[1.41, 2.37]
Child Only	1.13	1.06	N/A		1.11	1.08	1.06	1.02
	[0.80, 1.58]	[0.69, 1.63]			[0.85, 1.44]	[0.76, 1.53]	[0.93, 1.21]	[0.87, 1.19]
Sibling Only	**1.44*****	1.17	**1.71*****	0.90	**1.17*****	1.03	**1.18*****	1.04
	[1.24, 1.68]	[0.97, 1.42]	[1.05, 2.78]	[0.50, 1.62]	[1.08, 1.26]	[0.94, 1.14]	[1.09, 1.28]	[0.94, 1.15]

† $p<.10$, *$p<.05$, **$p<.01$, ***$p<.001$. Bold indicates a coefficient statistically significant at $p<0.10$. Note: BCL results for spouse and child losses are not included because participants did not have deceased spouses or children by age 17.

Table S9. Cox mixed-effects results with loss index at study entry predicting all-cause mortality during the study period, for all participants including those with no recorded losses.

	**Number of Deaths**		**Bereaved-dependent Childhood Losses (BCL)**		**Bereaved-dependent Life Stage (BLS)**		**Deceased-dependent Life Stage (DLS)**		**Deceased-dependent Life Stage (DLS) - exact**	
	**Unadjusted**	**Adjusted**	**Unadjusted**	**Adjusted**	**Unadjusted**	**Unadjusted**	**Unadjusted**	**Adjusted**	**Unadjusted**	**Adjusted**
Total sample	**1.12*****	**1.06*****	**1.27*****	1.06	**1.07*****	**1.03*****	**1.10*****	**1.05*****	**1.10*****	**1.05*****
	[1.09, 1.15]	[1.03, 1.10]	[1.16, 1.39]	[0.93, 1.20]	[1.05, 1.08]	[1.01, 1.04]	[1.08, 1.13]	[1.02, 1.08]	[1.08, 1.12]	[1.02, 1.07]
Race-stratified										
White	**1.13*****	**1.06*****	**1.27*****	1.01	**1.07*****	**1.03*****	**1.12*****	**1.06*****	**1.11*****	**1.04****
	[1.10, 1.16]	[1.03, 1.10]	[1.14, 1.18]	[0.87, 1.11]	[1.06, 1.09]	[1.01, 1.05]	[1.09, 1.15]	[1.02, 1.09]	[1.08, 1.14]	[1.01, 1.08]
Black	**1.15*****	**1.09***	**1.29****	1.23	**1.07*****	**1.05***	**1.11*****	**1.06†**	**1.10*****	**1.08***
	[1.08, 1.22]	[1.00, 1.20]	[1.07, 1.56]	[0.90, 1.69]	[1.05, 1.10]	[1.01, 1.09]	[1.06, 1.16]	[0.99, 1.14]	[1.04, 1.16]	[1.00, 1.17]
Hispanic	0.97	0.92	0.80	0.77	0.98	0.96	0.95	0.92	0.98	0.95
	[0.86, 1.10]	[0.79, 1.07]	[0.53, 1.21]	[0.46, 1.28]	[0.94, 1.04]	[0.89, 1.02]	[0.87, 1.04]	[0.82, 1.03[	[0.88, 1.09]	[0.83, 1.09]
Native	1.00	1.03	1.26	1.42	1.02	1.01	1.01	1.06	1.01	1.00
	[0.86, 1.17]	[0.82, 1.29]	P0.81, 1.96]	[0.62, 3.23]	[0.95, 1.09]	[0.91, 1.12]	[0.89, 1.14]	[0.89, 1.27]	[0.89, 1.14]	[0.82, 1.21]
Asian & Pacific	1.02	1.03	2.07	2.17	1.03	1.04	1.04	1.03	1.01	1.01
Islander	[0.74, 1.40]	[0.71, 1.50]	[0.84, 5.12]	[0.80, 5.84]	[0.90, 1.17]	[0.89, 1.21]	[0.80, 1.36]	[0.77, 1.40]	[0.78, 1.31]	[0.74, 1.37]
Other	0.99	1.16	0.79	1.10	0.98	1.04	0.96	1.12	0.99	1.11
	[0.82, 1.19]	[0.89, 1.52]	[0.39, 1.58]	[0.39, 3.11]	[0.90, 1.07]	[0.91, 1.18]	[0.83, 1.11]	[0.89, 1.40]	[0.84, 1.17]	[0.86, 1.42]
Relationship-stratified										
Parent Only	**1.12*****	**1.06*****	**1.29*****	1.06	**1.07*****	**1.03*****	**1.11*****	**1.05*****	**1.10*****	**1.05*****
	[1.09, 1.14]	[1.03, 1.09]	[1.15, 1.38]	[0.94, 1.21]	[1.05, 1.08]	[1.01, 1.04]	[1.08, 1.13]	[1.02, 1.08]	[1.08, 1.12]	[1.02, 1.08]
Spouse Only	1.06	**1.62****	N/A		1.16	**1.44******	1.09	**1.55****	**1.33†**	**1.60****
	[0.80, 1.38]	[1.21, 2.20]			[0.95, 1.42]	[1.17, 1.77]	[0.85, 1.40]	[1.19, 2.02]	[0.97, 1.83]	[1.14, 2.26]
Child Only	1.00	0.89	N/A		1.02	0.94	1.02	0.97	0.95	0.95
	[0.71, 1.40]	[0.57, 1.38]			[0.78, 1.33]	[0.69, 1.34]	[0.90, 1.16]	[0.82, 1.13]	[0.81, 1.13]	[0.79, 1.15]
Sibling Only	**1.35*****	1.06	**1.68***	0.85	**1.14****	0.99	**1.15*****	1.00	N/A	
	[1.15, 1.57	[0.87, 1.29]	[1.03, 2.76]	[0.47, 1.54]	[1.05, 1.23]	[0.90, 1.10]	[1.05, 1.24]	[0.90, 1.10]		

† *p*<.10, **p*<.05, ***p*< .01, ****p*< .001. Bold indicates a coefficient statistically significant at *p*< 0.10. Note: BCL results for spouse and child losses are not included because participants did not have deceased spouses or children by age 17. DLS exact results for sibling losses are not included because sibling age at death was not collected by HRS.

Table S10. Cox mixed-effects results for DLS index at study entry inclusive of losses with estimated age at death and restricted to losses with exact age at death predicting all-cause mortality during the study period.

	**Deceased-dependent Life Stage (DLS) index - estimated**		**Deceased-dependent Life Stage (DLS) index - exact**	
	**Unadjusted**	**Unadjusted**	**Unadjusted**	**Adjusted**
Total sample	**1.25*****<br>[1.22, 1.28]	**1.23*****<br>[1.19, 1.26]	**1.23*****<br>[1.21, 1.26]	**1.24*****<br>[1.20, 1.28]
Race-stratified				
White	**1.27*****<br>[1.24, 1.31]	**1.24*****<br>[1.20, 1.28]	**1.25*****<br>[1.21, 1.29]	**1.23*****<br>[1.19, 1.27]
Black	**1.22*****<br>[1.17, 1.29]	**1.21*****<br>[1.13, 1.31]	**1.20*****<br>[1.14, 1.27]	**1.26*****<br>[1.16, 1.37]
Hispanic	1.03<br>[0.94, 1.13]	0.98<br>[0.87, 1.11]	1.06<br>[0.94, 1.18]	1.06<br>[0.91, 1.24]
Native	**1.15***<br>[1.00, 1.32]	**1.24***<br>[1.02, 1.50]	**1.13†**<br>[0.98, 1.30]	1.15<br>[0.94, 1.41]
Asian & Pacific Islander	**1.46***<br>[1.05, 2.03]	1.32<br>[0.92, 1.89]	**1.38†**<br>[0.98, 1.93]	1.24<br>[0.86, 1.80]
Other	1.11<br>[0.96, 1.28]	**1.49****<br>[1.14, 1.95]	**1.16†**<br>[0.98, 1.39]	**1.46***<br>[1.08, 1.98]
Relationship-stratified				
Parent Only	**1.24*****<br>[1.21, 1.27]	**1.26*****<br>[1.22, 1.30]	**1.24*****<br>[1.21, 1.27]	**1.26*****<br>[1.22, 1.30]
Spouse Only	**1.38***<br>[1.08, 1.76]	**1.82*****<br>[1.41, 2.37]	**1.76*****<br>[1.28, 2.42]	**1.94*****<br>[1.38, 2.74]
Child Only	1.06<br>[0.93, 1.21]	1.02<br>[0.87, 1.19]	1.00<br>[0.85, 1.18]	1.01<br>[0.84, 1.21]
Sibling Only	**1.18*****<br>[1.09, 1.28]	1.04<br>[0.94, 1.15]	N/A	N/A

† *p*<.10, **p*<.05, ***p*< .01, ****p*< .001. Bold indicates a coefficient statistically significant at *p*< 0.10. Note: DLS exact results for sibling losses are not included because sibling age at death was not collected by HRS.

Table S11. Survey questions from Health and Retirement Study.

Variable	Wave	Dataset	Variable Name	Question
			**Losses**	
Parent loss	1992	W2PARS	V8201	Parent of the Section E respondent or the husband/partner?
			V8202	Mother or father?
			V8203	Is your (mother/father) living now?
			V8208	In what year did (she/he) die?
			V8209	How old was (she/he) when (she/he) died?
	1994	W2PARS	W8201	Is your (mother/father) and your spouse/partner's(mother/father) still living?
			W8205	In what year did (he/she) die?
			W8206	How old was (he/she) when (he/she) died?
	1996	h96d_h	E1557_1	Is your mother still living?
			E1557_2	Is your (husband's/wife's/partners/late husband's/late wife's/late partner's/late(husband's/partner's)/late (wife's/partners) mother still living?
			E1561_1, E1561_2	About how old was she?
			E1562_1, E1562_2, E1563_1, E1563_2	In what month and year did she die?
			E1566_1	Is your father still living?
			E1566_2A	Is your (husband's/wife's/partners/late husband's/late wife's/late partner's/late(husband's/partner's)/late (wife's/partners) father still living?
			E1570_1, E1570_2	About how old was he?
			E1571_1, E1571_2, E1572_1, E1572_2	In what month and year did he die?
	1998	h98d_h	F1906	Is your mother still living?
			F1912	About how old was she when she died?
			F1912, F1913	In what month and year did she die?
			F1916	Is your father still living?

			F1922	About how old was he when he died?
			F1922, F1923	In what month and year did he die?
	2000	h00d_h	G2122	Is your mother still living?
			G2127	About how old was she when she died?
			G2128, G2129	In what month and year did she die?
			G2132	Is your father still living?
			G2137	About how old was he when he died?
			G2138, G2139	In what month and year did he die?
	2002-2020	hXXf_r	YF001	Is your mother still living?
			YF006	About how old was she when she died?
			YF007, YF008	In what month and year did she die?
			YF011	Is your father still living?
			YF016	About how old was he when he died?
			YF017, YF018	In what month and year did he die?
Spouse loss	1992-1994	Not assessed		
	1996	h96cs_h	E227	Is (spouse/partner) still alive?
			E228, E229	In what month and year did (he/she) die?
	1998	h98cs_h	F504	Status (5 = Died)
			F505, F506	In what month and year did (he/she) die?
			F533, F535	In what month, day, and year was your (spouse) born?
	2000	h00cs_h	G545	Is (spouse) still alive?
			G546, G546	In what month and year did (he/she) die?
			G574, G576	In what month, day, and year was your (spouse) born?
	2002-2020	hXXa_h	YA023	Is (spouse) still alive?
			YA024, YA025	In what month and year did (spouse) die?
Child loss	1992	Not assessed		
	1994	W2KIDS	W8003	Relation to respondent
			W8004	Problem Code (3 = Died)
			W8005, W8006	In what month and year did (he/she) die?
	1996	h96d_mc<br>Date of death not assessed	E1365<br>E1367	Status (4 = Died)<br>Relationship to respondent
	1998	h98d_mc<br>Date of death not assessed	F1782<br>F1784	Status (4 = Died)<br>Relationship to respondent
	2000	h00d_mc<br>Date of death not assessed	G1998<br>G2000	Status (4 = Died)<br>Relationship to respondent
	2002-2020	hXXpr_mc	YX056_MC	Residency status (4 = Died)
			YZ057_MC	Previous wave residency status
			YX061_MC, YX063_MC	What is the relationship of (child) to (you/your current husband/wife/partner)?
			YX067_MC	What year (child) born?
			YX072_MC, YX073_MC	In what month and year did (child) die?
Sibling loss	1992	Not assessed		
	1994	W2SIBS	W8101	Relation to respondent/relation to spouse
			W8103	Problem code (3 = Died)
			W8104, W8105	In what month and year did (he/she) die?
	1996	h96pr_sb	E93	Updated sibling status (3 = Died)
			E159	Sibling relationship to respondent
		h96d_sb	E1644, E1645	In what month and year did (sibling) die?
	1998	h98pr_sb	F137	Status (3 = Died)
			F141	Relationship to respondent
		h98d_sb	F1991, F1992	In what month and year did (he/she) die?
	2000	h00pr_sb	G137	Status (3 = Died)
			G141	Relationship to respondent
		h00d_sb	G2217, G2218	In what month and year did (sibling) die?
	2002-2008	hXXpr_sb	YX056_SB	Residency status (4 = Died)
			YZ057_SB	Previous wave residency status
			YX061_SB, YX063_SB	What is the relationship of (sibling) to (you/your current husband/wife/partner)?
			YX067_SB	What year (sibling) born?
			YX072_SB, YX073_SB	In what month and year did (sibling) die?

		hXXf_sb	YF083, YF084	In what month and year did (sibling) die?
	2010-2012	hXXpr_sb<br><br>hXXf_sb	YZ249<br>YZ251<br>YF083, YF084	Residency status (4 = Died)<br>Relationship to respondent<br>In what month and year did (sibling) die?
	2014-2020	hXXpr_sb<br>Date of death not assessed	YZ249<br>YZ251	Residency status (4 = Died)<br>Relationship to respondent
			**Covariates and Outcomes**	
Participant age	All waves	RAND HRS Longitudinal	RABMONTH, RABYEAR	In what month and year were you born?
Participant mortality	All waves	RAND HRS Longitudinal	RADMONTH, RADYEAR	Respondent death month and year
Race	All waves	Cross-Wave Race and Ethnicity File (Restricted)	RACE2M1-RACE2M7	What race do you consider yourself to be: White, Black or African American, American Indian, Alaska Native, Asian, Native Hawaiian, Pacific Islander, or something else?
Sex	All waves	RAND HRS Longitudinal	RAGENDER	What is your sex?
Health-related quality of life	1994<br>1996<br>1998<br>2000<br>2002-	h94_w2b<br>h96b_r<br>h98b_r<br>h00b_r<br>hXXc_r	W301<br>E769<br>E1097<br>G1226<br>YC001	Would you say your health is excellent, very good, good, fair, or poor?
Parent educational attainment	All waves	RAND HRS Longitudinal	RAMEDUC<br>RAFEDUC	Mother's years of educational attainment<br>Father's years of educational attainment
Household capital income	All waves	RAND HRS Longitudinal	HZICAP	Sum of types of income from respondent and spouse
Childhood family SES	1992-1996<br>1998<br>2000<br>2002-2020	Not assessed<br>h98a_r<br>h00a_r<br>hXXb_r	 F993<br>G1080<br>YB020	Now think about your family when you were growing up, from birth to age 16. Would you say your family during that time was pretty well off financially, about average, or poor?
Age	All waves	RAND HRS Longitudinal	RZAGEY_B	Respondent age based on HHIDPN (also used for spouse and child age when their HHIDPN was available)
	All waves	RAND HRS Longitudinal	RZMOMAGE	Respondent's mother's age at death if deceased
	All waves	RAND HRS Longitudinal	RZDADAGE	Respondent's father's age at death if deceased
Household size	All waves	RAND HRS Longitudinal	HZHHRES	Other than you [and your (husband/wife/partner)], how many people are living with you?"
Number of times married	All waves	RAND HRS Longitudinal	RZMRCT	Number of times ever married
Number of living siblings	All waves	RAND HRS Longitudinal	RZLIVSIB	How many living sisters do you have?<br>How many living brothers do you have?
Number of children ever born	All waves	RAND HRS Longitudinal	RAEVBORN	How many children [have you fathered/have you given birth to]?
Census region	All waves	RAND HRS Longitudinal	RZCENREG	Census region of residence
Smoking	All waves	RAND HRS Longitudinal	RZSMOKEN	Do you smoke cigarettes now?
Drinking	1998-2020	RAND HRS Longitudinal	RZDRINK	Do you ever drink any alcoholic beverages, such as beer, wine, or liquor?
Chronic health conditions	All waves	RAND HRS Longitudinal	RZCONDE	Has a doctor ever told you that you have (condition)?

Note: The "XX" in the Dataset names names refers to the corresponding wave's year. For example, for the 2002 wave, replace "XX" with "02," whereas for the 2018 wave, replace "XX" with "18".
The first letter "Y" in the Variable names refers to the starting letter that corresponds with that wave according to HRS variable naming convention. For the 2002 wave, replace "Y" with "H"; for the 2004 wave, replace "Y" with "J"; for the 2006 wave, replace "Y" with "K", and so on.
The second letter "Z" in the Variable names for RAND files refers to the wave number. For the 1992 wave, replace "Z" with "1"; for the 1994 wave, replace "Z" with "2", and so on.

SI References

1. B. Bastian, B. Tejada Vera, E. Arias, Mortality trends in the United States, 1900–2018.
2. R. H. Weaver, C. Bolkan, J. D. Coatsworth, L. G. Hill, Introduction to the Special Issue: Toward a Lifespan Prevention Science - A Focus on Middle and Late Adulthood. *Prevention Science* **24**, 799-807 (2023).
3. U. Bronfenbrenner, P. A. Morris, "The Bioecological Model of Human Development" in Handbook of Child Psychology. (2007).
4. F. J. Infurna, D. Gerstorf, M. E. Lachman, Midlife in the 2020s: Opportunities and challenges. *American Psychologist* **75**, 470-485 (2020).
5. L. J. Luecken, D. S. Roubinov, Pathways to Lifespan Health Following Childhood Parental Death. *Social and Personality Psychology Compass* **6**, 243-257 (2012).
6. N. Lam *et al.*, The association between adverse childhood experiences and mental health, behaviour, and educational performance in adolescence: A systematic scoping review. *PLOS Mental Health* **1**, e0000165 (2024).
7. J. J. Arnett, R. Žukauskienė, K. Sugimura, The new life stage of emerging adulthood at ages 18-29 years: implications for mental health. *The Lancet Psychiatry* **1**, 569-576 (2014).
8. C. H. Rogers, F. J. Floyd, M. M. Seltzer, J. Greenberg, J. Hong, Long-term effects of the death of a child on parents' adjustment in midlife. *J Fam Psychol* **22**, 203-211 (2008).
9. M. E. Lachman, Mind the Gap in the Middle: A Call to Study Midlife. *Research in Human Development* **12**, 327-334 (2015).
10. M. E. Lachman, S. Teshale, S. Agrigoroaei, Midlife as a pivotal period in the life course: Balancing growth and decline at the crossroads of youth and old age. *International Journal of Behavioral Development* **39**, 20-31 (2014).
11. J. J. Arnett, Happily Stressed: The Complexity of Well-Being in Midlife. *Journal of Adult Development* **25**, 270-278 (2018).
12. R. Hutteman, M. Hennecke, U. Orth, A. K. Reitz, J. Specht, Developmental Tasks as a Framework to Study Personality Development in Adulthood and Old Age. *European Journal of Personality* **28**, 267-278 (2014).
13. R. O. Hansson, M. S. Stroebe, "Integrating Aging and Bereavement in Late Life". (American Psychological Association, Washington, DC, 2007), pp. 153-172.
14. D. Carr, H. Mooney, "Bereavement in later life" in Handbook of Aging and the Social Sciences (Ninth Edition), K. F. Ferraro, D. Carr, Eds. (Academic Press, 2021), pp. 239-254.
15. G. G. Fisher, L. H. Ryan, Overview of the Health and Retirement Study and Introduction to the Special Issue. *Work, Aging and Retirement* **4**, 1-9 (2017).
16. F. T. Juster, R. Suzman, An Overview of the Health and Retirement Study. *The Journal of Human Resources* **30**, S7-S56 (1995).
17. Sampling weights: Revised for tracker 2.0 & beyond, Health and Retirement Study (2019). https://hrs.isr.umich.edu/sites/default/files/biblio/wghtdoc_0.pdf (accessed 8 November 2024).
